# Common α-globin variants modify hematologic and other clinical phenotypes in sickle cell trait and disease

**DOI:** 10.1371/journal.pgen.1007293

**Published:** 2018-03-28

**Authors:** Laura M. Raffield, Jacob C. Ulirsch, Rakhi P. Naik, Samuel Lessard, Robert E. Handsaker, Deepti Jain, Hyun M. Kang, Nathan Pankratz, Paul L. Auer, Erik L. Bao, Joshua D. Smith, Leslie A. Lange, Ethan M. Lange, Yun Li, Timothy A. Thornton, Bessie A. Young, Goncalo R. Abecasis, Cathy C. Laurie, Deborah A. Nickerson, Steven A. McCarroll, Adolfo Correa, James G. Wilson, NHLBI Trans-Omics for Precision Medicine (TOPMed) Consortium, Hematology & Hemostasis, Diabetes, and Structural Variation TOPMed Working Groups, Guillaume Lettre, Vijay G. Sankaran, Alex P. Reiner

**Affiliations:** 1 Department of Genetics, University of North Carolina, Chapel Hill, North Carolina, United States of America; 2 Division of Hematology/Oncology, The Manton Center for Orphan Disease Research, Boston Children's Hospital, Harvard Medical School, Boston, Massachusetts, United States of America; 3 Department of Pediatric Oncology, Dana-Farber Cancer Institute, Harvard Medical School, Boston, Massachusetts, United States of America; 4 Program in Medical and Population Genetics, Broad Institute of MIT and Harvard, Cambridge, Massachusetts, United States of America; 5 Hematology, Department of Medicine, Johns Hopkins University, Baltimore, Maryland, United States of America; 6 Department of Medicine, Université de Montréal, Montréal, Quebec, Canada; 7 Montreal Heart Institute, Montréal, Quebec, Canada; 8 Stanley Center for Psychiatric Research, Broad Institute of MIT and Harvard, Cambridge, Massachusetts, United States of America; 9 Department of Genetics, Harvard Medical School, Boston, Massachusetts, United States of America; 10 Department of Biostatistics, University of Washington, Seattle, Washington, United States of America; 11 Department of Biostatistics, University of Michigan, Ann Arbor, Michigan, United States of America; 12 Department of Laboratory Medicine & Pathology, School of Medicine, University of Minnesota, Minneapolis, Minnesota, United States of America; 13 Zilber School of Public Health, University of Wisconsin-Milwaukee, Milwaukee, Wisconsin, United States of America; 14 Department of Genome Sciences, University of Washington, Seattle, Washington, United States of America; 15 Department of Medicine, University of Colorado Denver, Anschutz Medical Campus, Aurora, Colorado, United States of America; 16 Department of Biostatistics, University of North Carolina, Chapel Hill, North Carolina, United States of America; 17 Department of Computer Science, University of North Carolina, Chapel Hill, North Carolina, United States of America; 18 Division of Nephrology, Department of Medicine, University of Washington, Seattle, Washington, United States of America; 19 Seattle Epidemiologic Research and Information Center, Veterans Affairs Puget Sound Health Care System, Seattle, Washington, United States of America; 20 Department of Computational Medicine and Bioinformatics, University of Michigan, Ann Arbor, Michigan, United States of America; 21 Department of Medicine, University of Mississippi Medical Center, Jackson, Mississippi, United States of America; 22 Department of Physiology and Biophysics, University of Mississippi Medical Center, Jackson, Mississippi, United States of America; 23 Department of Epidemiology, University of Washington, Seattle, Washington, United States of America; Case Western Reserve University School of Medicine, UNITED STATES

## Abstract

Co-inheritance of α-thalassemia has a significant protective effect on the severity of complications of sickle cell disease (SCD), including stroke. However, little information exists on the association and interactions for the common African ancestral α-thalassemia mutation (−α3.7 deletion) and β-globin traits (HbS trait [SCT] and HbC trait) on important clinical phenotypes such as red blood cell parameters, anemia, and chronic kidney disease (CKD). In a community-based cohort of 2,916 African Americans from the Jackson Heart Study, we confirmed the expected associations between SCT, HbC trait, and the −α3.7 deletion with lower mean corpuscular volume/mean corpuscular hemoglobin and higher red blood cell count and red cell distribution width. In addition to the recently recognized association of SCT with lower estimated glomerular filtration rate and glycated hemoglobin (HbA1c), we observed a novel association of the −α3.7 deletion with higher HbA1c levels. Co-inheritance of each additional copy of the −α3.7 deletion significantly lowered the risk of anemia and chronic kidney disease among individuals with SCT (*P*-interaction = 0.031 and 0.019, respectively). Furthermore, co-inheritance of a novel α-globin regulatory variant was associated with normalization of red cell parameters in individuals with the −α3.7 deletion and significantly negated the protective effect of α-thalassemia on stroke in 1,139 patients with sickle cell anemia from the Cooperative Study of Sickle Cell Disease (CSSCD) (*P-*interaction = 0.0049). Functional assays determined that rs11865131, located in the major alpha-globin enhancer MCS-R2, was the most likely causal variant. These findings suggest that common α- and β-globin variants interact to influence hematologic and clinical phenotypes in African Americans, with potential implications for risk-stratification and counseling of individuals with SCD and SCT.

## Author summary

Recent work has shown that inheriting a single copy of the β-globin gene variant which causes sickle cell disease can be associated with medical risks, such as worsening kidney function. In individuals with sickle cell disease, co-inheritance of other globin gene variants, notably α-thalassemia, can modify an individual’s risk of clinical sequelae such as stroke. In this paper, our results suggest that inheritance of the same 3.7kb deletion that causes α-thalassemia in African populations can lower the risk of anemia and chronic kidney disease among African American community-dwelling individuals with sickle cell trait. Another α-globin genetic locus, located upstream within a well-known non-coding regulatory element, was found to modify associations of the α-thalassemia 3.7kb copy number variant with red blood cell traits in the African American general population and, in sickle cell disease patients, with risk of stroke. Using functional fine mapping and reporter assays, we localized rs11865131 and rs11248850 as the two most likely causal variants for these phenotypic associations. Additional molecular studies will be required to understand the complex regulatory mechanism by which these variants influence α-globin production.

## Introduction

Hemoglobin, the primary component of the red blood cell that carries oxygen to tissues in the body, is comprised of two β-globin chains, two α-globin chains, and a heme molecule bound to each subunit. In African Americans, β-globin protein structural variants, including hemoglobin S (HbS) and hemoglobin C (HbC), and α-globin copy number variants, such as the −α3.7 deletion, are common. Historically, carrier status (one copy) of these common α- and β-globin gene variants has been viewed as having no clinical implications or sequelae [1, 2]. Nevertheless, several well-powered studies have demonstrated that globin gene variants are associated with multiple erythrocyte parameters (i.e. hemoglobin, mean corpuscular volume (MCV), and mean corpuscular hemoglobin (MCH) [3]), and there is evidence that these variants at least partially contribute to the higher prevalence of anemia observed in African Americans compared to those of European ancestry [4–6]. More recently, other important clinical implications of globin variant carrier status have been identified. For example, HbS trait has been firmly established as a risk factor for kidney disease[7, 8], and the correlation between hemoglobin A1c (HbA1c) and fasting and 2-hour glucose measures can also be altered by sickle cell trait (SCT) status.[9]

In addition to being required for hemoglobin production in a 1-to-1 ratio, excess free α- or β-globin chains result in reduced red cell survival. However, the implications of and interactions among concurrent mutations in both globin genes are not fully understood. Several studies have investigated the effects of co-inheritance of one copy of the −α3.7 deletion on sickle cell disease or SCD (two copies of HbS), reporting that the −α3.7 deletion lowers the erythrocyte rheologic effects of hemoglobin S, which likely decreases the severity of many clinical complications of SCD[10–12], including stroke[13], priapism[14], and leg ulcers[15]. However, few studies have reported on the hematologic consequences of co-inheritance of SCT (one copy of HbS) and α-thalassemia trait (one copy of −α3.7)[16–18]. Moreover, whether presence of the −α3.7 deletion also ameliorates more recently recognized clinical sequelae of SCT, such as kidney function or HbA1c levels, is unknown.

In addition to copy number variants (CNVs) and coding variants, other common DNA polymorphisms located in the α and β -globin gene regions (chromosome 16p13.3 and 11p15.4, respectively) have been associated with red cell traits in large genome-wide association studies (GWAS).[19, 20] One such variant associated with erythrocyte traits is rs11248850 [19], which is located upstream of *HBA2*-*HBA1* within the major α-globin HS-40/ MCS-R2 regulatory enhancer element.[21, 22] Nevertheless, the exact functional variant(s) responsible for this signal have not yet been identified, and the effects of co-inheritance with −α3.7 copy number is unknown. In addition, the association of this regulatory variant on clinical complications of SCD has not been explored.

Whole genome sequencing (WGS) is unique in that it allows for the ascertainment of globin structural variants, noncoding genetic variants, and copy number variants simultaneously. Therefore, in a large, population-based sample of African Americans from the Jackson Heart Study (JHS), which was not selected based upon disease status or genotype, we performed WGS and confirmed previously reported associations and identified novel interactions between α-thalassemia carrier status (one copy), the GWAS sentinel variant rs11248850, and structural β-globin variants on red cell and clinical phenotypes. We performed functional fine-mapping of the genomic region containing the α-globin GWAS signal and identified the regulatory SNP rs11865131 as the most likely causal variant. Finally, in an independent cohort of SCD individuals, we examined the effect of co-inheritance of α-thalassemia and rs11865131 on important clinical phenotypes.

## Results

### Effect of structural and copy number globin variants on red cell phenotypes and clinical sequelae in JHS

The demographic and hematologic measures of the 2,916 JHS participants, overall and stratified by sex, are summarized in **S1 Table**. Anemia was present in 26%, microcytosis in 12%, and iron deficiency in 4% of individuals. Women were more likely to be anemic, microcytic, and iron deficient when compared to men. Overall, 67% of JHS participants had two diploid copies of the α3.7 CNV, 28% were heterozygous for the −α3.7 deletion, and 4% were homozygous for the −α3.7 deletion, but only 1% carried extra copies (3 or 4) (**S2 Table**). Overall, 9% of the African Americans in JHS were carriers of SCT and 3% were carriers of HbC trait.

The associations of α-globin 3.7 kb deletion status, HbS, and HbC trait on red cell and other clinically relevant quantitative phenotypes in JHS are shown in **Table 1.** Confirming the observations of previous reports, we observed that SCT, HbC trait, and each additional copy of the −α3.7 deletion were significantly associated with lower MCV and MCH, but higher RBC count and RDW; the −α3.7 deletion was associated with lower hemoglobin/ hematocrit, lower MCHC, and higher HbA1c levels; and HbC trait was associated with higher MCHC. There was no evidence that the α3.7 duplication was associated with any red cell parameter (**S3 Table**). We further confirmed that SCT was associated with lower eGFR and lower HbA1c in the JHS cohort [8, 9]. However, we observed an unexpected and novel association of the −α3.7 deletion with higher HbA1c levels. Consistent with the quantitative trait results, when hemoglobin, MCV, and eGFR were dichotomized and analyzed as binary traits, carrier status for the −α3.7 deletion, SCT, and HbC trait were each significantly associated with increased risk of microcytosis. Moreover, we observed that the −α3.7 deletion was associated with increased risk of anemia, while SCT was associated with increased risk of CKD (**Table 1**; **S4 Table**).

**Table 1:** Association of quantitative and binary red cell, kidney, and HbA1c phenotypes with hemoglobin S trait, hemoglobin C trait, and alpha-globin 3.7 kb deletion copy number.

Phenotype		Alpha globin −α3.7 deletion				Hemoglobin S		Hemoglobin C	
		One copy		Two copies					
***Quantitative traits***	**N**	Beta (SE)*	**p-value**	Beta (SE)*	**p-value**	Beta (SE)**	**p-value**	Beta (SE)**	**p-value**
**Hemoglobin (g/dL)**	2914	-0.398 (0.051)	<0.0001	-1.071 (0.123)	<0.0001	-0.160 (0.083)	0.054	-0.089 (0.144)	0.535
**Hematocrit (%)**	2914	-0.425 (0.148)	0.004	-1.375 (0.354)	<0.0001	-0.479 (0.236)	0.042	-0.797 (0.408)	0.051
**RBC Count (× 10**^**6**^ **cells/μl)**	2605	0.221 (0.019)	<0.0001	0.743 (0.046)	<0.0001	0.096 (0.032)	0.003	0.174 (0.053)	0.001
**MCV (fL)**	2605	-5.258 (0.233)	<0.0001	-15.295 (0.565)	<0.0001	-2.778 (0.442)	<0.0001	-4.876 (0.729)	<0.0001
**MCH (pg/dL)**	2605	-2.287 (0.088)	<0.0001	-6.340 (0.213)	<0.0001	-0.927 (0.173)	<0.0001	-1.206 (0.287)	<0.0001
**MCHC (%)**	2605	-0.626 (0.036)	<0.0001	-1.649 (0.0865)	<0.0001	-0.0016 (0.063)	0.980	0.491 (0.104)	<0.0001
**RDW (%)**	2604	0.365 (0.058)	<0.0001	0.927 (0.141)	<0.0001	0.308 (0.094)	0.001	0.595 (0.155)	<0.0001
**Ln HbA1c (%)**	2854	0.029 (0.007)	0.0001	0.031 (0.018)	0.090	-0.052 (0.012)	<0.0001	-0.021 (0.028)	0.447
**eGFR (mL/min/1.73 m**^**2**^**)**	2916	0.383 (0.775)	0.621	-3.522 (1.859)	0.058	-3.990 (1.234)	0.001	-1.714 (2.140)	0.423
***Binary outcomes***		OR (95%CI)*	**p-value**	OR (95%CI)*	**p-value**	OR (95%CI)**	**p-value**	OR (95%CI)**	**p-value**
**Anemia**	2914	1.779 (1.477, 2.142)	<0.0001	4.908 (3.259, 7.391)	<0.0001	1.244 (0.929, 1.664)	0.143	1.004 (0.593, 1.701)	0.988
**Microcytosis**	2605	4.186 (3.135, 5.586)	<0.0001	956.2 (229.5, 3983.3)	<0.0001	1.915 (1.330, 2.757)	0.0005	3.486 (2.088, 5.819)	<0.0001
**CKD**	2916	0.919 (0.720, 1.172)	0.495	1.420 (0.835, 2.414)	0.196	1.912 (1.363, 2.680)	<0.0001	1.157 (0.607, 2.204)	0.657

Abbreviations: RBC = red blood cell; MCV = mean corpuscular volume; MCH = mean corpuscular hemoglobin; MCHC = mean corpuscular hemoglobin concentration; RDW = red cell distribution width; HbA1c = glycated hemoglobin; eGFR = estimated glomerular filtration rate; OR = odds ratio; CI = confidence interval; CKD = chronic kidney disease.

*Beta coefficients (or ORs) correspond to estimates of the mean difference between (or risk associated with) carriers of the corresponding number of copies of the alpha-globin deletion compared to individuals carrying the normal diploid copy number. All models were adjusted for age, sex, and the first ten principal components of genetic ancestry.

**Beta coefficients (or ORs) correspond to estimates of mean difference between (or risk associated with) carriers of hemoglobin S trait or hemoglobin C trait compared to non-carriers. All models were adjusted for age, sex, and the first ten principal components of genetic ancestry.

### The −α3.7 deletion modifies the effect of SCT on red cell and clinical phenotypes

We next assessed the association of SCT and HbC trait, stratified by −α3.7 deletion copy number status, on red cell phenotypes, kidney function, and HbA1c. Lower hemoglobin levels are typically reported in SCT carriers, but we observed that co-inheritance of at least one copy of the −α3.7 deletion ameliorated this phenotype in SCT carriers (**Table 2**). Moreover, we only observed a higher risk of anemia for SCT carriers among individuals carrying the normal diploid copy number of the α-globin structural variant (odds ratio = 1.5, *P* = 0.02). In an interaction model, co-inheritance of −α3.7 significantly modified hemoglobin, RBC count, and anemia (interaction *P* values were ~0.03). Strikingly, we also observed that co-inheritance of the −α3.7 deletion attenuated the association of SCT with kidney dysfunction and HbA1c (**Table 2**). Specifically, the odds ratio of CKD associated with SCT was reduced from 2.6 among deletion non-carriers to 1.2 among deletion carriers and the interaction *P* values for HbA1c, eGFR, and CKD were 0.06, 0.04, and 0.02, respectively. We did not observe any modification of the effect of HbC on red cell traits by the −α3.7 deletion (**S5 Table**).

**Table 2:** Association of red cell and other clinically relevant phenotypes with sickle cell trait, stratified by number of copies of alpha-globin -3.7 kb deletion.

Red cell phenotype	No copies of −α3.7 deletion			1 copy of −α3.7 deletion			2 copies of −α3.7 deletion			*P*-value for genotype-genotype interaction
	N	Beta (SE) or OR (95%CI)*	p-value	N	Beta (SE) or OR (95%CI)*	p-value	N	Beta (SE) or OR (95%CI)*	p-value	
**Hemoglobin (g/dL)**	1991	-0.257 (0.101)	0.011	817	-0.003 (0.153)	0.982	106	0.543 (0.374)	0.150	0.035
**Hematocrit (%)**	1991	-0.715 (0.290)	0.014	817	-0.192 (0.440)	0.663	106	1.44 (1.22)	0.202	0.071
**RBC Count (× 10**^**6**^ **cells/μl)**	1786	0.0563 (0.0363)	0.122	727	0.0565 (0.0568)	0.320	92	0.422 (0.181)	0.022	0.032
**MCV (fL)**	1786	-2.679 (0.476)	<0.0001	727	-1.452 (0.636)	0.023	92	-2.570 (1.247)	0.043	0.787
**MCH (pg/dL)**	1786	-0.935 (0.181)	<0.0001	727	-0.310 (0.237)	0.191	92	-0.661 (0.421)	0.120	0.389
**MCHC (%)**	1786	-0.0386 (0.724)	0.594	727	0.199 (0.101)	0.050	92	0.237 (0.231)	0.308	0.121
**RDW (%)**	1785	0.352 (0.107)	0.001	727	0.088 (0.195)	0.653	92	0.399 (0.542)	0.463	0.477
**Anemia**	1991	1.527 (1.058, 2.202)	0.024	817	0.916 (0.530, 1.584)	0.754	106	0.555 (0.145, 2.124)	0.390	0.031
**Microcytosis**	1786	1.656 (0.852, 3.220)	0.137	727	1.976 (1.069, 3.650)	0.030	92	NA	NA	0.569
**Ln HbA1c (%)**	1952	-0.068 (0.014)	<0.0001	799	-0.029 (0.024)	0.228	103	0.035 (0.057)	0.536	0.059
**eGFR (mL/min/1.73 m**^**2**^**)**	1992	-5.40 (1.496)	0.0001	818	-2.626 (2.358)	0.266	106	9.358 (6.770)	0.170	0.042
**CKD**	1992	2.630 (1.743, 3.966)	<0.0001	818	1.15 (0.567, 2.315)	0.704	106	1.291 (0.209, 7.073)	0.784	0.019

Abbreviations: RBC = red blood cell; MCV = mean corpuscular volume; MCH = mean corpuscular hemoglobin; MCHC = mean corpuscular hemoglobin concentration; RDW = red cell distribution width; HbA1c = glycated hemoglobin; eGFR = estimated glomerular filtration rate; OR = odds ratio; CI = confidence interval; CKD = chronic kidney disease. NA = cannot be estimated due to small sample size.

*Beta coefficients (or ORs) correspond to estimates of mean difference between (or risk associated with) carriers of hemoglobin S trait compared to non-carriers. All models were adjusted for age, sex, and the first ten principal components of genetic ancestry.

### A novel α-globin regulatory variant in African Americans modifies the effect of α3.7 kb CNV on red cell parameters

**Fig 1:**
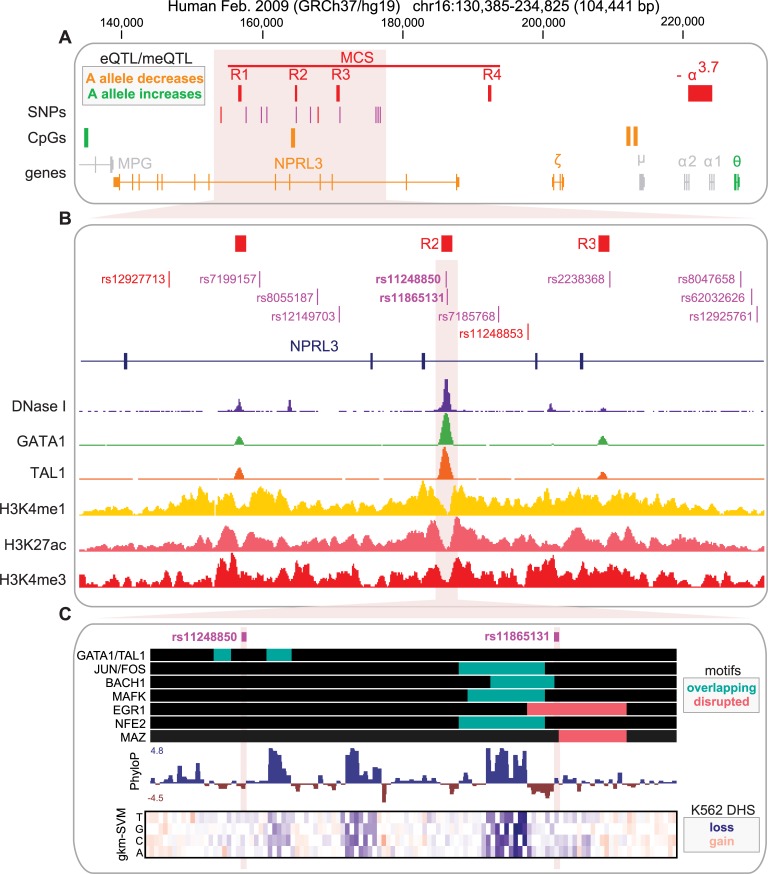
Genomic context of the α-globin regulatory GWAS locus. (**A-B**) The genomic region spanning the *NPRL3* and *HBA1* genes that contain the lead variant rs11248850 and its LD proxies, multi-species conserved sequences (MCS R1-4), and the -α3.7 deletion. eQTL(48, 49) and meQT(50) results from blood samples that reach genome-wide significance are shown, as well as the effect direction corresponding to the A alleles of rs11248850 and rs11865131. Although not illustrated, decreased expression of SNRNP2 was also associated in *cis* with the A allele. (**B**) rs11248850 and its LD proxies (magenta color indicates R^2^ ≥ 0.9 and red indicates R^2^ ≥ 0.8 but < 0.9). Signal tracks of DNaseI hypersensitivity(32), ChIP-seq for the key erythroid transcription factors GATA1 and TAL1(34), and histone modifications H3K4me1, H3K27ac and H3K4me3(33) from human derived erythroblasts are shown. The MCS-R2 enhancer element which overlaps rs11248850 and rs11865131 is highlighted in pink. (**C**) ChIP-seq occupancy sites are shown for transcription factors that were profiled in K562 cells and have motifs proximal to either of the MCS-R2 variants(43, 44). Evolutionary conservation across 100 vertebrates (PhyloP score)(47) and in silico mutagenesis(42) using a gkm-SVM trained on K562 DNase I hypersensitivity data identify likely functional motifs in the MCS-R2 element. Although rs11248850 appears to disrupt a GATA1 motif, it is not predicted by our software to have an effect as it resides in the low information content section between the GATA1 core and TAL1 core motifs.

In a recent meta-GWAS of red cell traits conducted in ~70,000 individuals of European or South Asian ancestry, a common single nucleotide variant (rs11248850, MAF = 0.50 in Europeans) was associated with MCH[19]. This variant, which is intronic to *NPRL3*, is located within the well-characterized major α-globin HS-40/ MCS-R2 enhancer element [22] approximately 70 kb upstream from the −α3.7 deletion (**Fig 1A and 1B**). In JHS African Americans, we observed an allele frequency of 0.22 for the rs11248850 A (variant) allele. The rs11248850 A allele frequency was higher among African American individuals carrying the normal diploid copy number of the α-globin 3.7 kb structural variant (0.27) compared to carriers of the −α3.7 deletion (0.14) (**S2 Table**). Nonetheless, the extent of linkage disequilibrium between rs11248850 and the α3.7 CNV was quite modest in JHS: the pairwise r^2^ between rs11248850 and the −α3.7 deletion was 0.04, and the pairwise r^2^ between rs11248850 and the α3.7 duplication was 0.0004.

Given the established associations of rs11248850 and the α-globin 3.7 kb CNV on red cell traits, we investigated whether the common variant could modify the effects of the α-globin CNV. In models adjusted for age, sex, and the first 10 principal components of genetic ancestry, we confirmed the previously reported association of the rs11248850 A allele with higher MCV, MCH, MCHC, and lower RBC count and RDW (**Table 3**). Interestingly, upon adjustment for α-globin 3.7 kb CNV status, the association of the rs11248850 A allele with red cell traits was largely abolished (**S6 Table**). To further explore the influence of the -α3.7 CNV on the association between rs11248850 and red cell traits, we performed analyses stratified by the presence or absence of the -α3.7 deletion (**Table 3**). Notably, the association of rs11248850 on RBC traits was almost exclusively confined to -α3.7 deletion carriers (**Table 3**; interaction *P* values range from 0.0001 to 0.03). Although we were unable to definitively determine if this modification occurred primarily in *cis* or in *trans* due to insufficient sample size, haplotype association analyses showed that the association of rs11248850 with RBC traits was apparent only when comparing rs11248850 minor allele effects on the background of the −α3.7 deletion allele (**S7 Table**).

**Table 3:** Association of red cell phenotypes with alpha-globin regulatory variant rs11248850, overall and by −α3.7 deletion status.

Red cell phenotype	Overall		No copy of −α3.7 deletion			≥1 copy of −α3.7 deletion			*P*-value for genotype-genotype interaction
	Beta (SE)*	p-value	N	Beta (SE)*	p-value	N	Beta (SE)*	p-value	
**Hemoglobin (g/dL)**	0.040 (0.039)	0.304	1991	-0.005 (0.045)	0.916	923	-0.117 (0.082)	0.154	0.308
**Hematocrit (%)**	-0.014 (0.111)	0.902	1991	-0.006 (0.130)	0.962	923	-0.403 (0.235)	0.086	0.201
**RBC Count (× 10**^**6**^ **cells/μl)**	-0.083 (0.015)	<0.0001	1786	-0.015 (0.016)	0.353	819	-0.152 (0.034)	<0.0001	0.0001
**MCV (fL)**	1.392 (0.207)	<0.0001	1786	0.167 (0.212)	0.431	819	1.719 (0.401)	<0.0001	0.0004
**MCH (pg/dL)**	0.558 (0.081)	<0.0001	1786	0.040 (0.080)	0.623	819	0.652 (0.153)	<0.0001	0.0002
**MCHC (%)**	0.117 (0.030)	0.0001	1786	-0.022 (0.032)	0.496	819	0.121 (0.058)	0.039	0.027
**RDW (%)**	-0.129 (0.058)	<0.0001	1785	-0.014 (0.047)	0.769	819	-0.268 (0.106)	0.012	0.010

Abbreviations: RBC = red blood cell; MCV = mean corpuscular volume; MCH = mean corpuscular hemoglobin; MCHC = mean corpuscular hemoglobin concentration; RDW = red cell distribution width; SE = standard error.

Models are adjusted for age, sex, and the first ten principal components of genetic ancestry.

*Beta coefficients correspond to estimates of the mean difference of the red cell parameter associated with carrying each additional copy of the rs11248850 A allele compared to the reference group of individuals carrying the rs11248850 G/G genotype.

### Functional fine-mapping of the α-globin regulatory locus

**Fig 2:**
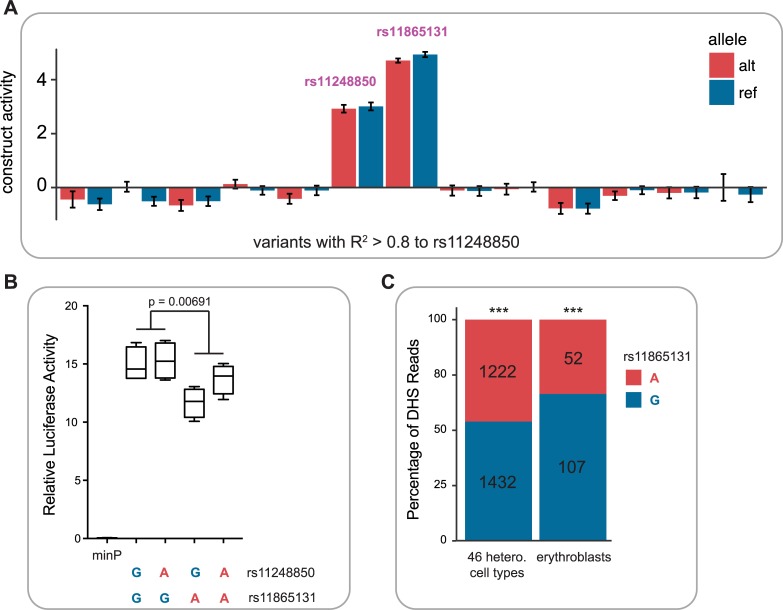
Functional assays for α-globin enhancer variants. Normalized activity (RNA barcode count divided by DNA barcode count) from the massively parallel reporter assay. The median of the entire library was set to 0; therefore an activity of 0 corresponds roughly to minP transcriptional levels. Only elements overlapping with the MCS-R2 element have robust regulatory activity in K562 cells. (**B**) Relative luciferase activity as compared to minP promoter for α-globin enhancer variants (rs11865131 and rs11248850). A significant allelic difference in enhancer activity is observed for rs11865131 (p = 6.91 x 10^−3^ for lower luciferase activity with A allele). (**C**) Allelic skew for rs11865131 from DNase I hypersensitivity (DHS) data in 46 heterozygous cell types previously identified in(37) and in erythroblasts only ***p<0.0001.

Given that a GWAS signal may represent tens or hundreds of variants in high linkage disequilibrium (LD), each of which could be causal, we set out to use genetic inheritance and functional assays to identify the most likely causal variant(s) underlying the α-globin proximal association. We identified 12 SNPs in high LD (r^2^ > 0.8 in JHS) with the sentinel GWAS variant rs11248850, spanning a ~25kb region of the *NPRL3* gene (**Fig 1A and 1B**). As none of these 13 variants were coding, we investigated the ability of elements containing each variant to regulate transcription in a massively parallel reporter assay[21]. Only elements containing the sentinel SNP rs11248850 or its proxy, rs11865131 (JHS r^2^ = 0.999 with predominant haplotypes of GG and AA), increased transcriptional activity of a reporter gene with a minimal promoter (**Fig 2A**). Importantly, these two elements are the only tested elements that overlap with open chromatin in erythroid cells, are part of the known MCS-R2 enhancer element, and overlap ChIP-Seq peaks for the key erythroid transcription factors GATA1 and TAL1 (**Fig 1B)**. Furthermore, in the highly homologous mouse locus, elegant chromatin confirmation assays have demonstrated that the MCS-R2 element interacts strongly with both α-globin gene promoters[23], and targeted genomic deletions of this element result in altered α-globin transcription[24]. In rare experiments of nature, humans specifically lacking only MCS-R2 exhibit decreased α-globin levels and altered red cell traits consistent with α-thalassemia trait[25, 26]; deletion of MCS-R2 in primary human hematopoietic stem cells caused knockdown of α-globin and restored globin chain balance in cells from β-thalassemia patients[27]. Thus, we reasoned that rs11248850 and rs11865131are strong candidate regulatory variants of the α-globin gene potentially underlying the original GWAS signal.

As our MPRA was technically only sensitive to large differences in activity (>1 log_2_-fold change [21]), we additionally performed individual allele-specific luciferase assays for all possible haplotypes of the two variants in the MCS-R2 element. Interestingly, rs11248850 did not show a significant difference in enhancer activity by allele, but its perfect proxy variant rs11865131 exhibited a significant allelic difference in enhancer activity (lower enhancer activity with the A allele, *P* = 0.00691) (**Fig 2B**). Consistent with these functional results, EIGEN-PC, a state of the art unsupervised method for identifying functional regulatory variants [28], predicts that rs11865131 is more likely to be functional than rs11248850 (5.92 vs. 2.20). Interestingly, these results were somewhat contrary to our expectation, given the closer proximity of rs11248850 to the binding sites for GATA1 and TAL1 (**Fig 1C**), two key erythroid transcription factors, which we have previously shown can be affected by regulatory variants even outside of their core motif [21]. To determine whether the lower transcriptional activity associated with the A allele could be observed endogenously in the genome, we investigated DNase I hypersensitivity (DHS) data across 46 cell lines (previously analyzed in [29]), including erythroblasts, and found significant evidence for allelic skew in the expected direction (*P* < 0.0001) **(Fig 2C)**. Indeed, this direction of effect is supported by two predictive algorithms, deepSEA [30] and gkmerSVM [31], trained on either K562 or CD34^+^ DHS datasets[21, 32]. Together, these data suggest that the most likely “causal” variant for this association lies within the MCS-R2 enhancer element, but further work, such as allelic replacement by genome editing across multiple independent cell clones, would be required to definitely prove causality for either rs11865131 or rs11248850.

To further investigate putative regulatory mechanisms by which the best candidate SNP, rs11865131, could alter regulatory activity, we investigated whether this variant affected any of the 426 transcription factor binding motifs in the HOCOMOCO database[33]. We found that the A allele was predicted to weaken the binding motifs for only ZNF219, MAZ, ZNF148, WT1, and EGR1/2. In K562 cells, we determined that both MAZ and EGR1 strongly occupied the MCS-R2 element (**Fig 1C**). Several other transcription factors, such as GATA1/TAL1, BACH1, and NFE2 occupied MCS-R2 and had motifs proximal to either rs11865131 or rs11248850, although these motifs were not predicted to be disrupted by either variant. The possibility that the GATA1/TAL1, BACH1, and NFE2 motifs could be important for regulation of this enhancer was supported by evolutionary conservation and by in silico mutagenesis using a gkm-SVM trained on K562 DHS (**Fig 1C**). To explore if any of these mechanisms were plausible *in vivo*, we investigated whether knockdown of 4 of these genes (MAZ, BACH1, TAL1, MAFK) in K562 cells would alter α-globin expression, but observed no significant changes in functional α-globin gene expression (**S1 Fig**). Therefore, the exact molecular mechanisms underlying the differences in expression observed in the reporter assay are currently unclear, but it is possible that rs11865131 could tune the binding of a TF such as NFE2 or JUN/FOS without disrupting the core motif (**Fig 1C**).

Thus far, our molecular investigation has not uncovered why the rs11865131-A allele, which is associated at the population level with higher MCV, MCH, and the amelioration of clinical phenotypes in SCT, shows *decreased* regulatory activity, when we expect that the A-allele should instead *increase* functional α-globin expression. To further investigate this, we turned to expression quantitative trait loci (eQTL) studies of whole blood. In most published blood eQTL studies, globin mRNAs were depleted[34], sample sizes were small[35], or microarray probe readings for the nearly identical adult α-globin genes (*HBA1* and *HBA2*) were of low quality[36]. Nevertheless, within a whole blood eQTL study of up to 5,311 individual adults[36, 37], we identified genome-wide significant associations for the rs11865131-A allele with decreased expression of the functional embryonic ζ-globin gene (*HBZ*), increased expression of the canonically non-functional θ-globin gene (*HBQ1*), and decreased expression for 2 proximal non-globin genes, *NPRL3* and *SNRNP2* (**Fig 1A**), all of which are highly expressed in cultured adult erythroblasts[38]. Furthermore, when we investigated a recent, large methylation QTL (meQTL) study of whole blood[39], the rs11865131-A allele was also associated with genome-wide significant changes in methylation (both increases and decreases) at 4 CpG dinucleotides that were measured within the α-globin region, including a CpG dinucleotide less than 300 base pairs away from the MCS-R2 element (**Fig 1A**). Taken all together, these analyses suggest that one or both of the SNPs identified within the MCS-R2 element are involved in the complex regulation of a spectrum of genes within the α-globin cluster, although the exact mechanisms remain to be elucidated.

### The α-globin regulatory variant rs11865131 modifies the protective effect of α-thalassemia on stroke in SCD patients

α-thalassemia status, which is most often due to the inheritance of the −α3.7 deletion, has been reported to influence both the severity of anemia and clinical sequelae of SCD (i.e. erythrocyte indices, risk of stroke, priapism, leg ulcers; reviewed in [40]). Given our finding that the MCS-R2 regulatory variant rs11865131 normalizes red cell parameters in African American carriers of the −α3.7 deletion, we explored the extent to which rs11865131 could modify clinical phenotypes in SCD patients with or without α-thalassemia (two copies of the −α3.7 deletion). We first confirmed that α-thalassemia status was protective against stroke, priapism, and leg ulcers in HbSS patients, although the protective effect was only significant for stroke[13–15] (OR = 0.45, *P* = 0.0004) (**Table 4**). Similar to our novel findings in JHS, we found that rs11865131/rs11248850 was associated with higher MCV and that this association was abolished by adjustment for α-thalassemia status (**S6 and S8 Tables**). Importantly, when we stratified these associations on the rs11865131 genotype, the protective effect of α-thalassemia on stroke was only present in rs11865131 GG homozygotes (OR = 0.29; *P* = 9.8 x 10^−4^), and there was no protective association of −α3.7 deletion with stroke among rs11865131 A-allele carriers (OR = 1.29; *P* = 0.47; interaction *P* = 0.0049). Similar stratum-specific association results were observed for priapism (protection in GG homozygotes only) but not for leg ulcers (**Table 4**).

**Table 4:** Association of alpha thalassemia with risk of clinical complications in the Cooperative Study of Sickle Cell Disease (CSSCD), stratified by α-globin rs11865131 genotype.

Complication	Overall				rs11865131-A Carriers				rs11865131-G homozygotes			
	OR (95%CI)	*P*	N HbSS/HbSS+α-thalassemia	N_affected_	OR (95%CI)	*P*	N HbSS/HbSS+α-thalassemia	N_affected_	OR (95%CI)	*P*	N HbSS/HbSS+α-thalassemia	N_affected_
**Stroke**	0.449 (0.288,0.701)	0.0004	1696/557	176 (152/24)	1.291 (0.648,2.575)	0.47	374/97	41 (30/11)	0.288 (0.137,0.604)	9.8 x 10^−4^	421/248	53 (44/9)
**Priapism**	0.704 (0.482,1.03)	0.07	801/273	205 (162/43)	0.982 (0.482,2)	0.96	172/51	41 (31/10)	0.554 (0.309,0.996)	0.048	207/115	60 (45/15)
**Leg ulcers**	0.750 (0.560,1.005)	0.054	1696/557	387 (300/87)	0.679 (0.369,1.248)	0.21	374/97	83 (68/15)	0.624 (0.376,1.035)	0.062	421/248	115 (76/39)

Abbreviations: OR = odds ratio; CI = confidence interval; N_affected_ = number of participants with the complication (number of HbSS participants with the complication / number of HbSS+α-thalassemia participants with the complication).

ORs correspond to estimates of risk difference between HbSS patients with α-thalassemia compared to HbSS patients without α-thalassemia. All models were adjusted for age, sex, and HbF levels.

## Discussion

Carrier status for hemoglobin chain variants, including the -α3.7 deletion and SCT, are common in African populations due to their strong selective advantage against severe malaria.[41] Although the implications of co-inheritance of α-thalassemia have been widely studied in SCD (two copies), genetic interactions of −α3.7 deletion, α-globin regulatory variants, and SCT (one copy) for clinical outcomes in the general African American population have not been fully elucidated. In a large community-based cohort of African Americans residing in the southeastern U.S., we now firmly demonstrate that co-inheritance of the −α3.7 deletion attenuates the risk of anemia and CKD in individuals with SCT. In addition, we find that a haplotype containing two common regulatory variants located within the α-globin HS-40/ MCS-R2 regulatory enhancer element normalizes red cell parameters in individuals with the −α3.7 deletion. Importantly, the same common haplotype negates the protective effect of α-thalassemia on stroke among HbSS patients from the Cooperative Study of Sickle Cell Disease (CSSCD). These findings suggest a complex relationship between hemoglobin variants and clinical phenotypes in African Americans.

Several recent studies have demonstrated that SCT is associated with progressive renal impairment including CKD, albuminuria, and end-stage renal disease.[7, 8] HbS-dependent sickling of erythrocytes in the hypoxic environment of the renal medulla has been theorized to result in renal injury; however the pathophysiology of renal disease in SCT remains largely unknown. In SCT, co-inheritance of α-thalassemia is a major determinant of intracellular HbS concentration, and lower HbS percentage due to increasing −α3.7 deletion copy number has been demonstrated to protect against urinary concentrating defects in individuals with SCT.[42] Our findings of a similar interaction between −α3.7 deletion and SCT for the development of CKD offers further biologic plausibility that HbS concentration is the causal determinant of SCT-related nephropathy. Furthermore, it is striking to note that individuals in JHS with coinheritance of −α3.7 deletion and SCT are nearly completely protected against CKD, whereas SCT carriers without α-thalassemia have a 2.6-fold increased risk of CKD. This finding may have important implications for SCT carrier risk stratification and clinical management.

SCT is associated with lower HbA1c measured using standard high-performance liquid chromatography assays[9], thereby limiting the clinical utility of HbA1c in screening and monitoring of glucose intolerance. In general, African Americans have higher HbA1c levels for the same level of fasting glucose compared to whites.[43] Our findings in JHS suggest that the −α3.7 deletion may account at least in part for the higher HbA1c levels among African Americans. Since the glycated residues of HbA1c reside on the N-terminus of the hemoglobin β-chain, the relative increase in the proportion of β-chain synthesis among α-thalassemia carriers may constitute a possible mechanism for the apparent increase in HbA1c. Similar to CKD and anemia, co-inheritance of α-thalassemia attenuated the HbA1c-lowering effect of SCT, suggesting that lower HbS percentage may be either associated with less interference with the HbA1c assay or results in improved erythrocyte survival.

α -globin gene expression is highly regulated by several multi-species conserved sequences (MCS-R) or enhancers located 30–70 kb upstream of *HBA1/2*.[44] In this region, we identified an association signal for red cell traits in our study of African Americans (previously reported in European populations[19]) that normalizes red cell parameters in individuals who carry the −α3.7 deletion and negates the protective effect of α-thalassemia for stroke in individuals with HbSS. By performing functional fine mapping with reporter assays and open chromatin localization in erythroid precursor cells, we identified rs11865131 within the MCS-R2 element as the most likely and rs11248850 as the second most likely causal variant for this association, but were unable to identify a specific mechanism by which variants comprising this common haplotype were likely to influence α-globin transcript. We were unable to fully resolve the paradox of how the rs11865131-A allele impairs enhancer activity, yet appears to be associated with laboratory and clinical parameters suggesting increased *HBA1*/*HBA2* expression, which ultimately suggests that regulation within this locus is quite complex and not fully understood. As an example of this emerging complexity, we and others recently discovered a low frequency, loss of function missense variant within *HBQ1* that was strongly associated with lower MCH[45], although *HBQ1* is largely thought to be a non-functional α-like globin gene. Here, the common rs11865131-A allele was associated with increased *HBQ1* but decreased expression of other proximal genes, including the functional embryonic globin gene *HBZ* and nearby *NPRL3*. Regardless of the exact regulatory mechanisms, our study suggests that this haplotype could be an important modifier of CKD, SCD, malaria[46], or other phenotypes whose severity is modified by the −α3.7 deletion. Indeed, we demonstrate that genotypes at rs11865131 can act as a “modifier of the modifier” in SCD patients, by impairing the protective effect of α-thalassemia on stroke risk. Furthermore, this variant may have an outsized effect on and contribute to the phenotypic variability of HbH, where transcriptional regulation of the single functional adult α-globin gene is paramount.[47]

The strengths of our study include the large, population-based cohort with WGS, which allowed assessment of α- and β-globin variant carrier status, including the α-globin 3.7kb CNV, in an unselected sample of African Americans with hematologic and other clinical data. By comparison with WGS, standard 1000 Genomes imputation from GWAS may not accurately infer genotype calls for the −α3.7 deletion, systematically underestimating the number of individuals with this deletion[3]. Our study does have limitations. We did not have hemoglobin electrophoresis data in JHS to determine HbS percentage, and our sample size was not large enough to evaluate subtle phenotypic differences between α-globin 3.7 CNV categories, particularly carriers of the α3.7 duplication. We also did not have statistical power to separate the diplotype effects of the risk allele rs11865131-A and the −α3.7 deletion. Many of our novel interaction findings would not meet a strict multiple testing threshold (for example, twelve interaction tests are performed between α-globin -3.7 kb deletion and sickle cell trait, giving a significance threshold of 0.05/12 = 0.004 for **Table 2** using a Bonferroni correction to adjust for multiple testing). Additionally, the current analysis does not assess the potential modifying influence of any additional HPFH/ β-globin single nucleotide variants or CNVs, though their frequency is unlikely to be appreciable in an unselected African American population-based sample. Finally, although we were able to strongly implicate two variants in the MCS-R2 element (rs11865131 and rs11248850) as responsible for altering gene expression in the α-globin cluster, we could not identify a definitive molecular mechanism by which either of these variants could act, and it is certainly possible that the observed enhancer activity may display context dependent activity.

In conclusion, in this large African American cohort, we show that co-inheritance of α-thalassemia significantly modified the risk of clinically relevant phenotypes such as CKD and anemia among individuals with SCT. We also demonstrate that α-globin regulatory variant rs11865131 is associated with decreased phenotypic expression of the −α3.7 deletion in both the general African American population and among patients with SCD. These findings may have important implications for future research and genetic counseling in African Americans with SCD and SCT.

## Methods

### Ethics statement

All Jackson Heart Study participants included in the analysis provided written informed consent for genetic studies. Approval was obtained from the institutional review board of the University of Mississippi Medical Center.

### Jackson Heart Study

The JHS is a prospective community-based study of African Americans in Jackson, Mississippi.[48, 49] During the baseline examination period (2000–2004) 5,306 self-identified African Americans were recruited from urban and rural areas of the three counties (Hinds, Madison and Rankin) that comprise the Jackson, Mississippi metropolitan area. Recruitment was limited to adult African Americans ≥ 21 years old. All participants included in analyses provided written informed consent for genetic studies. Approval was obtained from the institutional review board of the University of Mississippi Medical Center (UMMC).

### Phenotypic measurements in JHS

Data on participants’ health behaviors, medical history, and medication use were collected at baseline and subjects underwent venipuncture, including complete blood cell counts, measurements of iron indices, HbA1c, and serum creatinine. HbA1c was measured by NGSP-certified high-performance liquid chromatography (Tosoh 2.2). Serum creatinine (at baseline and exam 3) was measured using the Jaffé method and calibrated to measurements traceable to isotope-dilution mass spectrometry.[50] Estimated glomerular filtration rate (eGFR) was calculated using the CKD-EPI (CKD Epidemiology Collaboration) creatinine equation.[51] Chronic kidney disease (CKD) was defined as a creatinine eGFR < 60 mL/min/1.73 m^2^ at baseline or any follow-up visit[52]. Anemia was defined as hemoglobin level < 13 g/dL in men and < 12 g/dL in women.[53] Red cell microcytosis was defined as MCV < 80 fL. Iron deficiency was defined as ferritin < 15 ng/mL.

### Genotyping of α and β globin gene variants in JHS through NHLBI TOPMed

A total of 3,404 JHS participants underwent ~30X whole genome sequencing through the NHLBI TOPMed project. Inclusion in TOPMed was based on consent for widespread genetic data sharing, not phenotypic selection. Details of the sequencing, variant calling, and QC protocols are described in the **Supplemental Methods**. Genotypes for β-globin variants HbS (rs334) and HbC (rs33930165) and α-globin variant rs11248850 were extracted from the variant call set and used in association analyses. Principal components of genetic ancestry were calculated for each participant from the sequence data.[54] A subset of 3,009 JHS TOPMed participants underwent genotyping for the α-globin copy number variation (CNV) using the Genome STRiP multi-sample structural variant calling algorithm[55] and were eligible for the current analysis. We further excluded 6 individuals for low-quality CNV calls, 3 individuals who were homozygous and one individual missing data for the rs334 sickle cell variant, and 83 individuals who did not have hematologic phenotypes, leaving 2,916 individuals for analysis.

### Statistical analyses

The association of carrier status for α and β globin gene variants with hematologic quantitative traits was assessed using linear regression and reported as β regression coefficient (mean difference in red cell parameter between genotype comparison groups) and standard errors (SE). For binary traits (anemia, microcytosis, and CKD outcomes) logistic regression was used to estimate odds ratios and 95% confidence intervals (CI). Because of the small number of homozygotes for the α3.7 duplication (N = 2), we combined individuals carrying one or two extra copies of the α3.7 duplication in association analyses. All linear or logistic regression models were adjusted for age, sex, and the first 10 principal components of genetic ancestry to account for population stratification. To evaluate effect modification or genotype x genotype interactions, we performed association analyses stratified by α globin deletion status, and also introduced a multiplicative interaction term into regression models. All tests of main effect and effect modification were 2-sided and a *P* value < 0.05 was considered statistically significant. Haplotype association analyses were conducted using HAPSTAT (V3.0) [56].

### Functional fine-mapping of the alpha-globin regulatory region

We first identified all SNPs in high linkage disequilibrium (r^2^ > 0.8) with the previously reported sentinel GWAS variant rs11248850 from CEU and AFR populations of the 1000 Genomes Project Phase 3, and performed erythroid-specific functional annotations for each SNP, including genomic location, DNaseI hypersensitivity[57], histone modifications H3K4me1, H3K27ac and H3K4me3[58], and ChIP-seq for erythroid transcription factors GATA1 and TAL1.[59] Using a massively parallel reporter assay (MPRA),[21] 145 base pair elements centered at each allele of the rs11248850 sentinel SNP and all proxy SNPs were simultaneously tested for regulatory activity *in vitro*. Activity estimates from this assay are reported as previously described.[21] In the current study, for sites showing significant enhancer activity in the MPRA (rs11248850 and rs11865131), we additionally conducted allele-specific luciferase reporter assays assessing the function of MCS-R2 (436 nucleotides from chr16:163,406–163,841 in hg19) in K562 erythroid cells. The enhancer elements containing all combinations of allelic variants across rs11248850 and rs11865131 were cloned into the pGL4.24 minimal promoter (minP) containing vector (Promega). Dual luciferase assays in K562 erythroid cells were performed in a manner similar to assays assessing other similar non-coding regulatory variants in hematopoietic cells.[21, 60, 61] Allelic differences in enhancer activity were tested using a two-sided Student’s t-test. For the rs11865131 variant with a significant allelic difference, DNase I hypersensitivity (DHS) data from multiple cell lines (the sum of the counts for each allele across 46 heterozygous cell types) and erythroblasts were used to assess allelic skew in sequenced reads.[29, 62] EIGEN-PC, deepSEA, and gkm-SVM are algorithms that predict the function of non-coding variants and were used as previously described to *in silico* predict the effect of common variants.[21, 28, 30, 31] *In silico* mutagenesis was performed as described in [32].The ChIP-Atlas resource as well as K562 experiments from the ENCODE project were used to search for DHS and transcription factor (TF) occupancy in blood-based tissues.[63, 64] TF binding motif disruptions were determined using the motifbreakR R package [65] and the HOCOMOCO motif set.[33] PhyloP calculations for 100 vertebrates were accessed using the University of California Santa Cruz (UCSC) genome browser [66]. MAZ, BACH1, TAL1, and MAFK knockdown using siRNAs and subsequent RNA-seq was performed in duplicate or triplicate and gene expression was quantified as described previously by the ENCODE project (samples used were ENCFF253LFQ, ENCFF965QZJ, ENCFF133JRN, ENCFF289FNP, ENCFF595QDW, ENCFF848TKB, ENCFF675IJS, ENCFF064FJU, ENCFF464MQB, ENCFF517WDW, ENCFF213TLT, ENCFF634XCE, ENCFF714HMY, ENCFF035JUJ, ENCFF715DXD, ENCFF232WNR) [64]. eQTL [36, 37] and meQTL [39] results were obtained from the SMR data repository [67] and all genome-wide significant (p < 5.0x10^-8^) results for *cis* associations in the α-globin region are reported if identified in any single study.

### Effect of the A-allele of rs11865131 in sickle cell anemia (HbSS) patients with or without α-thalassemia

CSSCD has been described elsewhere [68, 69]. We used the clinical definitions from the CSSCD investigators to define incident stroke[13], priapism[14], and leg ulcers[15] in our analyses. The number of α-globin genes was determined by blot hybridization for 2,703 HbSS patients; participants with 2 or 3 copies of the α-globin genes were considered α-thalassemic.[70] Age at recruitment, sex, and fetal hemoglobin (HbF) levels were available for 2,253 of these 2,703 participants. CSSCD patients were genotyped on the Illumina 610-Quad array.[71] Genome-wide genotyping data was available for 1,140 of the 2,253 HbSS patients with α-thalassemia status information and covariates available. We imputed rs11865131 genotypes on 1000 Genomes Project (phase 3) haplotypes (version 5, hg19) using Minimac3 (v1.0.11) with high quality (imputation r^2^ = 0.995).

For baseline RBC traits, we tested the association with α-thalassemia status using linear regression correcting for sex, age, and the first 10 principal components of genetic ancestry. For dichotomous complications (stroke, priapism, and leg ulcers), we applied logistic regression, correcting for age, sex, and fetal hemoglobin levels. We further stratified these analyses by presence of the rs11865131 A-allele. Adjustment for the first 10 principal components did not change the conclusions from these analyses of dichotomous complication measures.

## Supporting information

S1 Fig siRNA knockdown of MAZ, BACH1, TAL1, and MAFK. Mean RNA-seq gene expression from 2–3 replicates each for control siRNA (WT) and siRNAs targeting MAZ (siMAZ), BACH1 (siBACH1), TAL1 (siTAL1), and MAFK (siMAFK). Partial knockdown was verified for target genes of between ~20–47%. None of the 3 canonically functional α-globin genes (HBA1, HBA2, HBZ) were significantly altered by MAZ knockdown (log_2_ fold change < 0.33).
(EPS)

S1 Table Demographic characteristics and hematologic traits of Jackson Heart Study participants. Abbreviations: N, number; SD, standard deviation; RBC = red blood cell; MCV = mean corpuscular volume; MCH = mean corpuscular hemoglobin; MCHC = mean corpuscular hemoglobin concentration; RDW = red cell distribution width. Anemia was defined as hemoglobin level less than 13 g/dL in men and less than 12 g/dL in women; microcytosis was defined as MCV less than 80 fL; iron deficiency was defined as ferritin less than 15 ng/mL.
(PDF)

S2 Table Distribution of alpha globin 3.7 copy number by alpha- and beta-globin variant genotypes (N = 2,916). (PDF)

S3 Table Association of red cell phenotypes with ≥1 copy of 3.7 kb alpha-globin duplication. Abbreviations: MCV = mean corpuscular volume; MCH = mean corpuscular hemoglobin; MCHC = mean corpuscular hemoglobin concentration; RDW = red cell distribution width; OR = odds ratio; CI = confidence interval. NA = cannot be estimated due to small sample size. *Beta coefficients (or ORs) correspond to estimates of the mean difference between (or risk associated with) carriers of one or more copies of the alpha-globin duplication compared to individuals carrying the normal diploid copy number. All models were adjusted for age, sex, and the first ten principal components of genetic ancestry.
(PDF)

S4 Table Case-control analysis of anemia and microcytosis outcomes, according to alpha-globin 3.7 kb deletion copy number. Abbreviations: OR = odds ratio; CI = confidence interval; CKD = chronic kidney disease.*ORs correspond to estimates of the mean difference between (or risk associated with) carriers of the corresponding number of copies of the alpha-globin deletion compared to individuals carrying the normal diploid copy number. All models were adjusted for age, sex, and the first ten principal components of genetic ancestry.
(PDF)

S5 Table Association of red cell traits with hemoglobin C trait, stratified by number of copies of alpha-globin -3.7 kb deletion. Abbreviations: RBC = red blood cell; MCV = mean corpuscular volume; MCH = mean corpuscular hemoglobin; MCHC = mean corpuscular hemoglobin concentration; RDW = red cell distribution width; OR = odds ratio; CI = confidence interval. NA = cannot be estimated due to small sample size. *Beta coefficients (or ORs) correspond to estimates of mean difference between (or risk associated with) carriers of hemoglobin C trait compared to non-carriers. All models were adjusted for age, sex, and the first ten principal components of genetic ancestry.
(PDF)

S6 Table Association of red cell phenotypes with alpha-globin regulatory variant rs11248850, with and without adjustment for α–globin copy number. Abbreviations: RBC = red blood cell; MCV = mean corpuscular volume; MCH = mean corpuscular hemoglobin; MCHC = mean corpuscular hemoglobin concentration; RDW = red cell distribution width; SE = standard error. Model A is minimally adjusted for age, sex, and the first 10 principal components of genetic ancestry. Model B is adjusted for age, sex, the first 10 principal components of genetic ancestry, and also α–globin copy number genotype. *Beta coefficients correspond to estimates of the mean difference of the red cell parameter associated with carrying each additional copy of the rs11248850 A allele compared to the reference group of individuals carrying the rs11248850 G/G genotype.
(PDF)

S7 Table Alpha globin haplotype association analysis with red cell traits. (PDF)

S8 Table Association of α-thalassemia and rs11865131 with RBC traits in the Cooperative Study of Sickle Cell Disease (CSSCD). Abbreviations: RBC = red blood cell; MCV = mean corpuscular volume; MCH = mean corpuscular hemoglobin; SE: Standard Error; OR = odds ratio; CI = confidence interval. For the α-thalassemia model, beta coefficients correspond to estimates of mean difference between HbSS patients without α-thalassemia compared to HbSS patients with α-thalassemia. For rs11865131, beta coefficients correspond to estimates of mean difference associated with carrying each additional copy of the rs11865131 A-allele compared to the reference group of individuals carrying the rs11865131 G/G genotype. All models were adjusted for age, sex, and the first 10 principal components of genetic ancestry.
(PDF)

S1 File Supplemental methods. **Sequencing and Data Processing Methods- Freeze 4,** Published with permission of the TOPMed Publications Committee. From https://www.nhlbiwgs.org/sequencing-and-data-processing-methods-freeze4 (link available only to TOPMed investigators, information copied here).
(DOCX)

S2 File The trans-omics in precision medicine program (TOPMed). Published with permission of the TOPMed Publications Committee.
(DOCX)
