# Supplementary material for: Common α-globin variants modify hematologic and other clinical phenotypes in sickle cell trait and disease
Source: PLoS Genet. 2018 Mar 28;14(3):e1007293. doi: 10.1371/journal.pgen.1007293 (PMC5891078; doi:10.1371/journal.pgen.1007293)
Supplement: S1 Table — Abbreviations: N, number; SD, standard deviation; RBC = red blood cell; MCV = mean corpuscular volume; MCH = mean corpuscular hemoglobin; MCHC = mean corpuscular hemoglobin concentration; RDW = red cell distribution width. Anemia was defined as hemoglobin level less than 13 g/dL in men and less than 12 g/dL in women; microcytosis was defined as MCV less than 80 fL; iron deficiency was defined as ferritin less than 15 ng/mL. (PDF) [file pgen.1007293.s002.pdf]

S1 Table. Demographic characteristics and hematologic traits of Jackson Heart Study participants.

Trait		Overall		Women			Men		
	N	Mean ± SD or N (%)	Range	N	Mean ± SD or %	Range	N	Mean ± SD or %	Range
**Age (years)**	2916	55 ± 13	21-93	1844	56 ± 13	21-88	1072	55 ± 13	21-93
**Current Smoker**	2891	385 (13%)		1826	188 (10%)		1075	197 (19%)	
**Hemoglobin (g/dL)**	2914	13.0 ± 1.5	3.0-19.2	1843	12.4 ± 1.2	3.0-16.9	1071	14.1± 1.3	8.7-19.2
**Hematocrit (%)**	2914	39.3 ± 4.3	11.9-57.2	1844	37.6 ± 3.4	11.9-52.2	1070	42.1 ± 3.8	26.2-57.2
**RBC Count**	2605	4.54 ± 0.52	2.91-6.68	1647	4.36 ± 0.43	2.91-6.35	958	4.84 ± 0.51	2.92-6.68
**MCV (fL)**	2605	86.9 ± 6.4	57.6-114.2	1647	86.5 ± 6.5	57.6-112.3	958	87.7 ± 6.0	62.3-114.2
**MCH (pg/dL)**	2605	28.9 ± 2.5	15.9-38.9	1647	28.7 ± 2.6	15.9-38.9	958	29.2 ± 2.3	19.8-37.5
**MCHC (%)**	2605	33.2 ± 0.90	27.7-36.0	1647	33.1 ± 0.92	27.7-36.0	958	33.3 ± 0.86	30.6-36.0
**RDW (%)**	2604	13.7 ± 1.3	11.1-27.7	1646	13.8 ± 1.4	11.1-24.1	958	13.6 ± 1.3	11.3-27.7
**Anemia**	2914	750 (25.7%)		1843	559 (30.3%)		1071	191 (17.8%)	
**Microcytosis**	2605	310 (11.9%)		1647	218 (13.2%)		958	92 (9.6%)	
**Iron deficiency**	2897	105 (3.6%)		1873	95 (5.2%)		1092	10 (0.9%)	

Abbreviations: N, number; SD, standard deviation; RBC=red blood cell; MCV = mean corpuscular volume; MCH = mean corpuscular hemoglobin; MCHC = mean corpuscular hemoglobin concentration; RDW = red cell distribution width.

Anemia was defined as hemoglobin level less than 13 g/dL in men and less than 12 g/dL in women; microcytosis was defined as MCV less than 80 fL; iron deficiency was defined as ferritin less than 15 ng/mL.
