# Supplementary material for: Common α-globin variants modify hematologic and other clinical phenotypes in sickle cell trait and disease
Source: PLoS Genet. 2018 Mar 28;14(3):e1007293. doi: 10.1371/journal.pgen.1007293 (PMC5891078; doi:10.1371/journal.pgen.1007293)
Supplement: S2 Table — (PDF) [file pgen.1007293.s003.pdf]

**S2 Table. Distribution of alpha globin 3.7 copy number by alpha- and beta-globin variant genotypes (N=2,916).**

| Number of copies of α3.7 variant | α-globin rs11248850 genotype | | | β-globin rs334 genotype (sickle cell trait) | | β-globin rs33930165 genotype (Hb C trait) | | Total |
|---|---|---|---|---|---|---|---|---|
| | G/G | G/A | A/A | HbA/A | HbA/S | HbA/A | HbA/C | |
| **0 (−α3.7/−α3.7)** | 101 (6%) | 4 (0.4%) | 1 (0.6%) | 93 (3%) | 13 (5%) | 101 (4%) | 5 (6%) | **106 (4%)** |
| **1 (−α3.7/αα3.7)** | 578 (33%) | 223 (22%) | 17 (10%) | 749 (28%) | 69 (28%) | 797 (28%) | 21 (27%) | **818 (28%)** |
| **2 (αα3.7/αα3.7)** | 1065 (61%) | 758 (76%) | 144 (88%) | 1799 (67%) | 167 (67%) | 1914 (67%) | 52 (67%) | **1966 (67%)** |
| **3 (ααα3.7/αα3.7)** | 12 (0.7%) | 11 (1%) | 1 (0.6%) | 23 (0.9%) | 1 (0.4%) | 24 (0.9%) | 0 (0%) | **24 (0.8%)** |
| **4 (ααα3.7/ααα3.7)** | 1 (0.1%) | 1 (0.1%) | 0 (0%) | 2 (0.1%) | 0 (0%) | 2 (0.1%) | 0 (0%) | **2 (0.1%)** |
| **Total** | **1757 (60%)** | **997 (34%)** | **162 (6%)** | **2666 (91%)** | **250 (9%)** | **2838 (97%)** | **78 (3%)** | **2916 (100%)** |
