# Supplementary material for: Common α-globin variants modify hematologic and other clinical phenotypes in sickle cell trait and disease
Source: PLoS Genet. 2018 Mar 28;14(3):e1007293. doi: 10.1371/journal.pgen.1007293 (PMC5891078; doi:10.1371/journal.pgen.1007293)
Supplement: S3 Table — Abbreviations: MCV = mean corpuscular volume; MCH = mean corpuscular hemoglobin; MCHC = mean corpuscular hemoglobin concentration; RDW = red cell distribution width; OR = odds ratio; CI = confidence interval. NA = cannot be estimated due to small sample size. *Beta coefficients (or ORs) correspond to estimates of the mean difference between (or risk associated with) carriers of one or more copies of the alpha-globin duplication compared to individuals carrying the normal diploid copy number. All models were adjusted for age, sex, and the first ten principal components of genetic ancestry. (PDF) [file pgen.1007293.s004.pdf]

S3 Table. Association of red cell phenotypes with ≥1 copy of 3.7 kb alpha-globin duplication.

**Red cell phenotype**	**N**	**Beta (SE) or OR (95%CI)***	**p-value**
**Hemoglobin (g/dL)**	2914	0.169 (0.243)	0.485
**Hematocrit (%)**	2914	0.394 (0.700)	0.573
**RBC Count (x 10^6 cells/μl)**	2605	0.003 (0.088)	0.970
**MCV (fL)**	2605	0.806 (1.0857)	0.458
**MCH (pg/dL)**	2605	0.405 (0.409)	0.322
**MCHC (%)**	2605	0.168 (0.166)	0.313
**RDW (%)**	2604	-0.479 (0.271)	0.077
**Anemia (OR, 95% CI)**	2914	0.619 (0.209, 1,837)	0.388
**Microcytosis (OR, 95%CI)**	2605	NA	NA

Abbreviations: MCV = mean corpuscular volume; MCH = mean corpuscular hemoglobin; MCHC = mean corpuscular hemoglobin concentration; RDW = red cell distribution width; OR = odds ratio; CI = confidence interval. NA = cannot be estimated due to small sample size.

*Beta coefficients (or ORs) correspond to estimates of the mean difference between (or risk associated with) carriers of one or more copies of the alpha-globin duplication compared to individuals carrying the normal diploid copy number. All models were adjusted for age, sex, and the first ten principal components of genetic ancestry.
