# Supplementary material for: Common α-globin variants modify hematologic and other clinical phenotypes in sickle cell trait and disease
Source: PLoS Genet. 2018 Mar 28;14(3):e1007293. doi: 10.1371/journal.pgen.1007293 (PMC5891078; doi:10.1371/journal.pgen.1007293)
Supplement: S4 Table — Abbreviations: OR = odds ratio; CI = confidence interval; CKD = chronic kidney disease.*ORs correspond to estimates of the mean difference between (or risk associated with) carriers of the corresponding number of copies of the alpha-globin deletion compared to individuals carrying the normal diploid copy number. All models were adjusted for age, sex, and the first ten principal components of genetic ancestry. (PDF) [file pgen.1007293.s005.pdf]

S4 Table. Case-control analysis of anemia and microcytosis outcomes, according to alpha-globin 3.7 kb deletion copy number.

Outcome	No copies of −α3.7 deletion			1 copy of −α3.7 deletion			2 copies of −α3.7 deletion		
	N total	N cases	OR (95% CI)	N total	N cases	OR (95% CI)	N total	N cases	OR (95% CI)
**Anemia**	1991	491 (21%)	ref	817	268 (33%)	1.8 (1.5, 2.1)	106	68 (58%)	4.9 (3.3, 7.4)
**Microcytosis**	1786	90 (5%)	ref	727	130 (18%	4.2 (3.1, 5.6)	92	90 (98%)	956 (230, 3983)
**CKD**	1992	311 (16%)	ref	818	130 (16%)	0.9 (0.7, 1.2)	106	22 (21%)	1.4 (0.8, 2.4)

Abbreviations: OR = odds ratio; CI = confidence interval; CKD=chronic kidney disease.

*ORs correspond to estimates of the mean difference between (or risk associated with) carriers of the corresponding number of copies of the alpha-globin deletion compared to individuals carrying the normal diploid copy number. All models were adjusted for age, sex, and the first ten principal components of genetic ancestry.
