# Supplementary material for: Common α-globin variants modify hematologic and other clinical phenotypes in sickle cell trait and disease
Source: PLoS Genet. 2018 Mar 28;14(3):e1007293. doi: 10.1371/journal.pgen.1007293 (PMC5891078; doi:10.1371/journal.pgen.1007293)
Supplement: S5 Table — Abbreviations: RBC = red blood cell; MCV = mean corpuscular volume; MCH = mean corpuscular hemoglobin; MCHC = mean corpuscular hemoglobin concentration; RDW = red cell distribution width; OR = odds ratio; CI = confidence interval. NA = cannot be estimated due to small sample size. *Beta coefficients (or ORs) correspond to estimates of mean difference between (or risk associated with) carriers of hemoglobin C trait compared to non-carriers. All models were adjusted for age, sex, and the first ten principal components of genetic ancestry. (PDF) [file pgen.1007293.s006.pdf]

S5 Table. Association of red cell traits with hemoglobin C trait, stratified by number of copies of alpha-globin -3.7 kb deletion.

Red cell phenotype	No copies of −α3.7 deletion			1 copy of −α3.7 deletion			2 copies of −α3.7 deletion			
	N	Beta (SE) or OR (95%CI)	p-value	N	Beta (SE) or OR (95%CI)	p-value	N	Beta (SE) or OR (95%CI)	p-value	*P*-value for genotype-genotype interaction
**Hemoglobin (g/dL)**	1991	-0.074 (0.175)	0.674	817	0.008 (0.269)	0.977	106	-0.559 (0.541)	0.305	0.787
**Hematocrit (%)**	1991	-0.886 (0.504)	0.079	817	-0.283 (0.775)	0.715	106	-2.509 (1.609)	0.122	0.996
**RBC Count**	1786	0.165 (0.059)	0.005	727	0.195 (0.096)	0.042	92	-0.247 (0.340)	0.022	0.961
**MCV (fL)**	1786	-5.190 (0.768)	<0.0001	727	-3.957 (1.065)	<0.0001	92	-1.553 (2.329)	0.507	0.339
**MCH (pg/dL)**	1786	-1.213 (0.294)	<0.0001	727	-1.081 (0.398)	0.007	92	-0.393 (0.779)	0.615	0.833
**MCHC (%)**	1786	0.603 (0.116)	<0.0001	727	0.295 (0.171)	0.085	92	0.088 (0.424)	0.835	0.067
**RDW (%)**	1785	0.577 (0.174)	0.001	727	0.651 (0.328)	0.048	92	0.142 (0.991)	0.887	0.928
**Anemia (OR, 95% CI)**	1991	1.082 (0.543, 2.156)	0.822	817	0.546 (0.190, 1.572)	0.262	106	5.472 (0.516, 57.99)	0.158	0.859
**Microcytosis (OR, 95% CI)**	1786	4.653 (2.217, 9.763)	<0.0001	727	5.094 (2.010, 12.91)	0.001	NA	NA	NA	0.585

Abbreviations: RBC=red blood cell; MCV = mean corpuscular volume; MCH = mean corpuscular hemoglobin; MCHC = mean corpuscular hemoglobin concentration; RDW = red cell distribution width; OR = odds ratio; CI = confidence interval. NA = cannot be estimated due to small sample size.

*Beta coefficients (or ORs) correspond to estimates of mean difference between (or risk associated with) carriers of hemoglobin C trait compared to non-carriers. All models were adjusted for age, sex, and the first ten principal components of genetic ancestry.
