# Supplementary material for: Common α-globin variants modify hematologic and other clinical phenotypes in sickle cell trait and disease
Source: PLoS Genet. 2018 Mar 28;14(3):e1007293. doi: 10.1371/journal.pgen.1007293 (PMC5891078; doi:10.1371/journal.pgen.1007293)
Supplement: S6 Table — Abbreviations: RBC = red blood cell; MCV = mean corpuscular volume; MCH = mean corpuscular hemoglobin; MCHC = mean corpuscular hemoglobin concentration; RDW = red cell distribution width; SE = standard error. Model A is minimally adjusted for age, sex, and the first 10 principal components of genetic ancestry. Model B is adjusted for age, sex, the first 10 principal components of genetic ancestry, and also α–globin copy number genotype. *Beta coefficients correspond to estimates of the mean difference of the red cell parameter associated with carrying each additional copy of the rs11248850 A allele compared to the reference group of individuals carrying the rs11248850 G/G genotype. (PDF) [file pgen.1007293.s007.pdf]

S6 Table. Association of red cell phenotypes with alpha-globin regulatory variant rs11248850, with and without adjustment for α–globin copy number.

		Model A		Model B	
**Red cell phenotype**	**N**	**Beta (SE)***	**p-value**	**Beta (SE)***	**p-value**
**Hemoglobin (g/dL)**	2914	0.040 (0.039)	0.304	-0.046 (0.039)	0.237
**Hematocrit (%)**	2914	-0.014 (0.111)	0.902	-0.119 (0.113)	0.295
**RBC Count**	2605	-0.083 (0.015)	<0.0001	-0.033 (0.014)	0.021
**MCV (fL)**	2605	1.392 (0.207)	<0.0001	0.281 (0.178)	0.115
**MCH (pg/dL)**	2605	0.558 (0.081)	<0.0001	0.085 (0.067)	0.209
**MCHC (%)**	2605	0.117 (0.030)	0.0001	-0.011 (0.027)	0.698
**RDW (%)**	2604	-0.129 (0.058)	<0.0001	-0.057 (0.044)	0.202

Abbreviations: RBC=red blood cell; MCV = mean corpuscular volume; MCH = mean corpuscular hemoglobin; MCHC = mean corpuscular hemoglobin concentration; RDW = red cell distribution width; SE= standard error.

Model A is minimally adjusted for age, sex, and the first 10 principal components of genetic ancestry. Model B is adjusted for age, sex, the first 10 principal components of genetic ancestry, and also α–globin copy number genotype.

*Beta coefficients correspond to estimates of the mean difference of the red cell parameter associated with carrying each additional copy of the rs11248850 A allele compared to the reference group of individuals carrying the rs11248850 G/G genotype.
