# Supplementary material for: Common α-globin variants modify hematologic and other clinical phenotypes in sickle cell trait and disease
Source: PLoS Genet. 2018 Mar 28;14(3):e1007293. doi: 10.1371/journal.pgen.1007293 (PMC5891078; doi:10.1371/journal.pgen.1007293)
Supplement: S7 Table — (PDF) [file pgen.1007293.s008.pdf]

S7 Table. Alpha globin haplotype association analysis with red cell traits.

				Hemoglobin		Hematocrit		RBC count		MCH		MCHC		MCV		RDW	
Haplo	Freq	rs11248850	3.7 kb indel	Beta (SE)	p	Beta (SE)	p	Beta (SE)	p	Beta (SE)	p	Beta (SE)	p	Beta (SE)	p	Beta (SE)	p
H1	0.61	G	0	ref	-	-	-	-	-	-	-	-	-	-	-	-	-
H2	0.17	G	1	-0.40 (0.05)	<0.001	-0.42 (0.14)	0.002	0.27 (0.02)	<0.001	-2.7 (0.08)	<0.001	-0.72 (0.02)	<0.001	-6.3 (0.21)	<0.001	0.03 (0.004)	<0.001
H3	0.21	A	0	-0.008 (0.04)	0.84	-0.05 (0.12)	0.67	-0.02 (0.02)	0.37	0.04 (0.07)	0.57	-0.02 (0.03)	0.62	0.13 (0.20)	0.49	-0.002 (0.003)	0.57
H4	0.009	A	1	-1.4 (0.32)	<0.001	-2.4 (1.04)	0.02	-0.06 (0.10)	0.56	-2.3 (0.38)	<0.001	-0.58 (0.17)	0.0009	-5.1 (1.04)	<0.001	0.004 (0.02)	0.84
H2 v. H4					0.001		0.07		0.001		NS		NS		NS		0.12
