# Supplementary material for: Common α-globin variants modify hematologic and other clinical phenotypes in sickle cell trait and disease
Source: PLoS Genet. 2018 Mar 28;14(3):e1007293. doi: 10.1371/journal.pgen.1007293 (PMC5891078; doi:10.1371/journal.pgen.1007293)
Supplement: S8 Table — Abbreviations: RBC = red blood cell; MCV = mean corpuscular volume; MCH = mean corpuscular hemoglobin; SE: Standard Error; OR = odds ratio; CI = confidence interval. For the α-thalassemia model, beta coefficients correspond to estimates of mean difference between HbSS patients without α-thalassemia compared to HbSS patients with α-thalassemia. For rs11865131, beta coefficients correspond to estimates of mean difference associated with carrying each additional copy of the rs11865131 A-allele compared to the reference group of individuals carrying the rs11865131 G/G genotype. All models were adjusted for age, sex, and the first 10 principal components of genetic ancestry. (PDF) [file pgen.1007293.s009.pdf]

S8 Table. Association of α-thalassemia and rs11865131 with RBC traits in the Cooperative Study of Sickle Cell Disease (CSSCD).

	α-thalassemia			rs11865131			rs11865131 adjusted for α-thalassemia		
**Trait**	**Beta (SE)**	***P***	**N HbSS/HbSS+α-thalassemia**	**Beta (SE)**	***P***	**N**	**Beta (SE)**	***P***	**N**
**RBC Count (x 10^6 cells/µl)**	0.424 (0.036)	1.17×10^{-29}	716/318	-0.040 (0.030)	0.181	1034	0.020 (0.029)	0.490	1034
**MCV (fL)**	-7.543 (0.492)	6.41×10^{-48}	716/318	1.349 (0.419)	0.001	1034	0.309 (0.386)	0.425	1034
**MCH (pg/dL)**	-2.693 (0.177)	1.02×10^{-46}	611/307	0.238 (0.158)	0.133	918	-0.163 (0.144)	0.255	918
**Hematocrit (%)**	1.566 (0.241)	1.29×10^{-10}	789/342	-0.184 (0.189)	0.330	1131	0.041 (0.189)	0.827	1131
**Hemoglobin (g/dL)**	0.352 (0.077)	5.62×10^{-6}	789/342	-0.060 (0.060)	0.318	1131	-0.010 (0.060)	0.871	1131

Abbreviations: RBC=red blood cell; MCV = mean corpuscular volume; MCH = mean corpuscular hemoglobin; SE: Standard Error; OR = odds ratio; CI = confidence interval.

For the α-thalassemia model, beta coefficients correspond to estimates of mean difference between HbSS patients without α-thalassemia compared to HbSS patients with α-thalassemia. For rs11865131, beta coefficients correspond to estimates of mean difference associated with carrying each additional copy of the rs11865131 A-allele compared to the reference group of individuals carrying the rs11865131 G/G genotype. All models were adjusted for age, sex, and the first 10 principal components of genetic
