# Supplementary material for: Common α-globin variants modify hematologic and other clinical phenotypes in sickle cell trait and disease
Source: PLoS Genet. 2018 Mar 28;14(3):e1007293. doi: 10.1371/journal.pgen.1007293 (PMC5891078; doi:10.1371/journal.pgen.1007293)
Supplement: S1 File — Sequencing and Data Processing Methods- Freeze 4, Published with permission of the TOPMed Publications Committee. From https://www.nhlbiwgs.org/sequencing-and-data-processing-methods-freeze4 (link available only to TOPMed investigators, information copied here). (DOCX) [file pgen.1007293.s010.docx]

**Supplemental Methods**

Sequencing and Data Processing Methods- Freeze 4, Published with permission of the TOPMed Publications Committee

From https://www.nhlbiwgs.org/sequencing-and-data-processing-methods-freeze4 (link available only to TOPMed investigators, information copied here)

**Overview**

Trans-Omics for Precision Medicine (TOPMed), sponsored by the National Heart, Lung and Blood Institute (NHLBI), generates scientific resources to enhance our understanding of fundamental biological processes that underlie heart, lung, blood and sleep disorders (HLBS). It is part of the broader Precision Medicine Initiative, which aims to provide disease treatments that are tailored to an individual’s unique genes and environment. TOPMed contributes to this initiative by integrating whole-genome sequencing (WGS) and other –omics data (e.g., metabolic profiles, protein and RNA expression patterns) with molecular, behavioral, imaging, environmental, and clinical data. In doing so, the TOPMed program seeks to uncover factors that increase or decrease the risk of disease, identify subtypes of disease, and develop more targeted and personalized treatments.

Currently, TOPMed includes 60 different studies with ~120,000 samples undergoing whole genome sequencing. The studies encompass several experimental designs (e.g. cohort, case-control, family) and many different clinical trait areas (e.g. asthma, COPD, atrial fibrillation, atherosclerosis, sleep). See study descriptions under the “Studies” tab on this web site.

The following sections of this document describe methods of data acquisition, processing and quality control (QC) for TOPMed WGS data contained in the April 2017 releases. Briefly, ~30X whole genome sequencing was performed at several different Sequencing Centers (named in the Table). All samples for a given study were sequenced at the same center, except for a small number of control samples described below. The reads were aligned to human genome build GRCh37 at each center using similar, but not identical, processing pipelines. The resulting BAM files were transferred from all centers to the TOPMed Informatics Research Center (IRC), where they were re-aligned to build GRCh37, using a common pipeline to produce a set of ‘harmonized’ BAM files. Both the Sequencing Center-specific BAM and the harmonized BAM files were deposited in the NCBI Sequence Read Archive (SRA), where they were converted to ‘.sra’ file format. Both center-specific and IRC-harmonized .sra files are available to users with approved access to a given study. The IRC performed joint genotype calling on all samples in the April 2017 releases. The resulting VCF files were split by study and consent group for distribution to approved dbGaP users, but can be reassembled easily for cross-study, pooled analysis because the files for all studies contain identical lists of variant sites. Quality control was performed at each stage of the process by the Sequencing Centers, the IRC and the TOPMed Data Coordinating Center (DCC). Only samples and variants that passed QC are included in the genotype call sets distributed with the April 2017 releases.

Sequence/genotype data files provided in the April 2017 dbGaP releases include the following:

1. Aligned read data for each sample in ‘.sra’ format (which is readily convertible to BAM format). Each sample has two .sra files: one from the Sequencing Center and the other from the IRC
2. Genotype call sets (one per chromosome) in ‘.vcf’ format

**TOPMed DNA sample/sequencing-instance identifiers**

Each DNA sample processed by TOPMed was given a unique identifier as “NWD” followed by six digits (e.g. NWD123456). These identifiers are unique across all TOPMed studies. Each NWD identifier is associated with a single study subject identifier used in other dbGaP files (such as phenotypes, pedigrees and consent files). A given subject identifier may link to multiple NWD identifiers when duplicate samples are taken from the same individual. Study investigators assigned NWD IDs to subjects, and their biorepositories assigned DNA samples/ NWD IDs to specific bar-coded wells/tubes supplied by their Sequencing Center, and recorded those assignments in a sample manifest, along with other metadata (e.g. sex, DNA extraction method). At each Sequencing Center, the NWD ID was propagated through all phases of the pipeline and is the primary identifier in all results files. Each NWD ID resulted in a single sequencing instance (i.e. ‘run’ in SRA terminology), which is linked to a single subject identifier in the sample-subject mapping file for each accession. Note that subject identifiers are study-specific and may not be unique across all of TOPMed accessions.

**Control Samples**

One parent-offspring trio from the Framingham Heart Study (FHS) was sequenced at each of four Sequencing Centers (family ID 746, subject IDs 13823, 15960 and 20156). All four WGS runs for each subject are provided in the TOPMed FHS accession (phs000974). In addition, HapMap subjects NA12878 (CEU, Lot K6) and NA19238 (YRI, Lot E2) were sequenced at each of the Sequencing Centers in alternation, once approximately every 1000 study samples. The HapMap sequence data will be released publicly as a BioProject.

The average pairwise non-reference genotype discordance rate among 69 pairs of duplicate sequenced samples is 5 x 10-5. The genotype discordance rate is very sensitive to the stringency level used for variant site filtering. This low figure is evidence of the benefit of 30x whole genome sequencing and suggests that the current filtering threshold suitably balances sensitivity and specificity. It must be acknowledged that these 27 control samples were among 4,047 duplicate and related samples which provided a negative training set for the SVM classifier used for site level filtering (see Variant Filtering section).

To calculate non-reference discordance, the genotypes of each DNA sample are called independently from separate sets of sequence reads, often from different Sequencing Centers. The denominator for each pairwise comparison is the number of sites where at least one of the two samples has a non-reference genotype called (either het or hom-alt). The numerator is the number of sites where the two genotypes disagree.

**Sequencing Center Methods**

Northwest Genomics Center

Deborah Nickerson

**DNA Sample Handling and QC**

The NWGC centralizes all receipt, tracking, and quality control/assurance of DNA samples in a Laboratory Information Management System. Samples are assigned unique barcode tracking numbers and have a detailed sample manifest (i.e., identification number/code, sex, DNA concentration, barcode, extraction method). Initial QC entails DNA quantification, sex typing, and molecular “fingerprinting” using a high frequency, cosmopolitan genotyping assay. This ‘fingerprint’ is used to identify potential sample handling errors and provides a unique genetic ID for each sample, which eliminates the possibility of sample assignment errors. In addition, we spot check ~8% of the samples per batch on an agarose gel to check for high molecular weight DNA, if DNA degradation is detected all samples are checked. Samples are failed if: (1) the total amount, concentration, or integrity of DNA is too low; (2) the fingerprint assay produces poor genotype data or (3) sex-typing is inconsistent with the sample manifest. Barcoded plates were shipped to Macrogen for library construction and sequencing.

**Library Construction**

Libraries were constructed with a minimum of 0.4ug gDNA and are prepared in Covaris 96 microTUBE plates and sheared through a Covaris LE220 focused ultrasonicator targeting 350 bp inserts. The resulting sheared DNA is selectively purified using sample purification beads to make the precise length of insert; End-repair (repaired to blunt end), A-tailing (A-base is added to 3’end), and ligation (Y-shaped adapter is used which includes a barcode) are performed as directed by TruSeq PCR-free Kit (Illumina, cat# FC-121-3003) protocols. A second Bead cleanup is performed after ligation to remove any residual reagents and adapter dimers. To verify the size of adapter-ligated fragments, we validate the template size distribution by running on a 2200 TapeStation (Agilent, Catalog # G2964AA) using a TapeStation DNA Screen Tape (Agilent, Catalog 5067-5588). The final libraries are quantified by qPCR assay using KAPA library quantification kit (cat.# KK4808 and KK4953) on a Light Cycler 480 instrument(Roche, cat# 05015278001).

**Clustering and Sequencing**

Eight normalized and indexed libraries were then pooled together and denatured before cluster generation on a cBot. The 8-plex pools were loaded on eight lanes of a flow cell and sequenced on a HiSeqX using illumina’s HiSeq X ten reagents kit (V2.5, cat# FC-501-2521). For cluster generation, every step is controlled by cBot. When cluster generation is complete, the clustered patterned flow cells are then sequenced with sequencing software HCS (HiSeq Control Software). The runs are monitored for %Q30 bases using the SAV (Sequencing Analysis Viewer). Using RTA 2 (Real Time Analysis 2) the BCLs (base calls) were de-multiplexed into individual FASTQs per sample using illumina package bcl2fastq v2.15.0 and transferred from Macrogen to NWGC for alignment, merging, variant calling and sequencing QC.

**Read Processing**

Our processing pipeline consists of aligning FASTQ files to a human reference (hs37d5) using BWA-MEM (Burrows-Wheeler Aligner; v0.7.10) (Li and Durbin 2009). All aligned read data are subject to the following steps: (1) “duplicate removal” is performed, (i.e., the removal of reads with duplicate start positions; Picard MarkDuplicates; v1.111) (2) indel realignment is performed (GATK IndelRealigner; v3.2) resulting in improved base placement and lower false variant calls and (3) base qualities are recalibrated (GATK BaseRecalibrator; v3.2). Sample BAM files were “squeezed” using Bamutil with default parameters and checksummed before being transferred to the IRC.

**Sequence Data QC**

All sequence data undergo a QC protocol before they are released to the TOPMed IRC for further processing. For whole genomes, this includes an assessment of: (1) mean coverage; (2) fraction of genome covered greater than 10x; (3) duplicate rate; (4) mean insert size; (5) contamination ratio; (6) mean Q20 base coverage; (7) Transition/Transversion ratio (Ti/Tv); (8) fingerprint concordance > 99%; and (9) sample homozygosity and heterozygosity. All QC metrics for both single-lane and merged data are reviewed by a sequence data analyst to identify data deviations from known or historical norms. Lanes/samples that fail QC are flagged in the system and can be re-queued for library prep (< 1% failure) or further sequencing (< 2% failure), depending upon the QC issue.

**Informatics Research Center Methods**

Tom Blackwell, Hyun Min Kang and Goncalo Abecasis

Center for Statistical Genetics, Department of Biostatistics, University of Michigan

The IRC pipeline consists of two major processes diagrammed in the figure below: (1) Harmonization of data from the BAM files provided by the Sequencing Centers and (2) joint variant discovery and genotype calling across studies. Detailed protocols for these processes are given in the following sections.


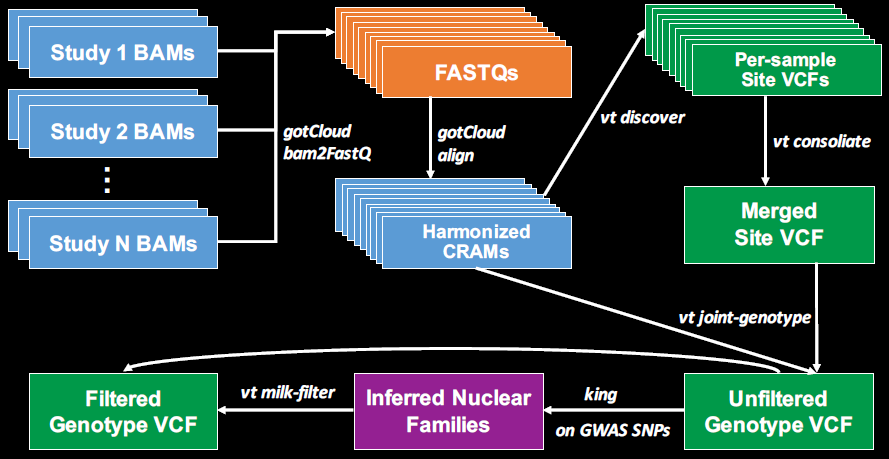


**Harmonization of Read Alignments**

Prior to joint variant discovery and genotype calling by the IRC, the sequence data obtained from the TOPMed Sequencing Centers are remapped using a standard protocol to produce “harmonized” BAM files.

Sequence data are received from each sequencing center in the form of .bam files mapped to the 1000 Genomes hs37d5 build 37 decoy reference sequence. File transfer is via Aspera or Globus Connect, depending on the center. Batches of 100 - 500 .bam files in a single directory are convenient, along with a file of md5 checksums for the files in that directory.

The IRC validates the md5 checksum, indexes each .bam file using ‘samtools index’ and runs local programs Qplot (Li, et al, 2013, doi:10.1155/2013/865181) and verifyBamId (Jun, et al, 2012, doi:10.1016/j.ajhg.2012.09.004) for incoming sequence quality control. We make a backup copy in .cram format using ‘samtools view -C’ with base call quality scores reduced to 8 bins using ‘bamUtils squeeze’ (if not already binned at the sequencing center). We add ‘’NWD’’ DNA sample identifiers to the read group header lines (Illumina) and convert from UCSC to Ensembl chromosome names (Illumina and Macrogen) using ‘samtools reheader’. In house scripts add read group tags as needed to legacy Illumina sequencing data from 2012-2013.

To produce the ‘’harmonized’’ read mappings which are deposited in dbGaP and used for variant discovery and genotyping, we remap the sequence data in each .bam file to the 1000 Genomes hs37d5 decoy reference sequence using a uniform ‘’IRC standard’’ protocol. This uses ‘bamUtils bam2fastq’ with flags ‘--splitRG --gzip’ to extract all sequence reads by read group into paired-end .fastq format, then remaps to hs37d5.fa using ‘bwa mem’ version 0.7.12-r1039 with ‘-M’ to mark split alignments as secondary. Read group header information is copied from the sequencing center .bam file. Followed by ‘samtools sort’, ‘bamUtils polishBam’, ‘bamUtils mergeBam’ and ‘bamUtils dedup_LowMem --recab’ with flags ‘--binMid --binQualS 2,3,10,20,25,30,35,40,50 --maxBaseQual 44’ to recalibrate and bin base call quality scores. Samtools version 1.2 is used throughout. Processing is coordinated and managed by our ‘GotCloud’ processing pipeline.

Description of our local and standard software tools is available from:

- http://genome.sph.umich.edu/wiki/BamUtils
- http://genome.sph.umich.edu/wiki/GotCloud
- http://genome.sph.umich.edu/wiki/QPLOT
- http://www.htslib.org (samtools)
- https://github.com/lh3/bwa (bwa, current)
- http://bio-bwa.sourceforge.net (bwa, outdated)

Software sources:

- http://genome.sph.umich.edu/w/images/7/70/BamUtilLibStatGen.1.0.13.tgz
- https://github.com/statgen/bamUtil/releases/tags/v1.0.13
- https://github.com/statgen/gotcloud/releases/tags/gotcloud.1.17.4
- http://www.sph.umich.edu/csg/zhanxw/software/qplot/qplot-source.20130627.tar.gz
- https://github.com/statgen/verifyBamId/releases/tags/v1.1.2
- https://github.com/samtools/samtools/releases/download/1.2/samtools-1.2.tar.bz2
- https://sourceforge.net/projects/bio-bwa/files/bwakit/bwakit-0.7.12_x64-linux.tar.bz2
- https://github.com/lh3/bwa (source code)

GRCh37 genome reference source:

ftp://ftp.1000genomes.ebi.ac.uk/vol1/ftp/technical/reference/phase2_reference_assembly_sequence/hs37d5.fa.gz

(*Note that the original 1000 Genomes .fasta file is razip compressed. For use with the current version of samtools, this file must be expanded and (optionally) re-compressed with bgzip to support the current samtools faidx indexing.)*

The two sequence quality criteria we use in order to pass sequence data on for joint variant discovery and genotyping are: estimated DNA sample contamination below 3%, and fraction of the genome covered at least 10x 95% or above. DNA sample contamination is estimated from the sequencing center read mapping using software verifyBamId (Goo Jun, et al. (2012) Detecting and estimating contamination of human DNA samples in sequencing and array based genotype data. American Journal of Human Genetics, v.91, n.5, pp.839-848).

The IRC-harmonized BAM files and the original BAM files from the Sequencing Centers are deposited in the NCBI Sequence Read Archive, where they are stored in ‘.sra’ format. These files can be accessed by approved users through the dbGaP “Run Selector”. Note that the two different read mappings (IRC-harmonized versus Sequencing Center) can be distinguished in the Run Selector by the column ‘’Alignment Provider’’. Users download .sra files using the SRA ToolKit. Each sample is identified to the toolkit with an ID starting with "SRR", which can be matched with the sample ID (NWD ID) using the dbGaP Run Selector. Users can request all data for a sample, or just a specific region of the genome, and easily convert from SAM back to BAM format using samtools. The SRA ToolKit documentation includes an example of how to create a BAM file for a specified genomic region for a given sample.

- SRA ToolKit setup: https://trace.ncbi.nlm.nih.gov/Traces/sra/sra.cgi?view=toolkit_doc&f=std
- SRA ToolKit .sra to .bam for specified region: https://trace.ncbi.nlm.nih.gov/Traces/sra/sra.cgi?view=toolkit_doc&f=sam-dump
- samtools: <http://www.htslib.org/>

**Variant Discovery and Genotype Calling**

**Overview**

The genotype call sets provided for the dbGaP accessions released in April 2017 are from “freeze 4” of the variant calling pipeline performed by the TOPMed Informatics Research Center (Center for Statistical Genetics, University of Michigan, Hyun Min Kang, Tom Blackwell and Gonçalo Abecasis). The software tools used in this version of the pipeline are available in the following repository: https://github.com/statgen/topmed_freeze4_calling. The following description refers to specific components of the pipeline. The variant calling software tools are under active development; updated versions can be accessed at http://github.com/atks/vt or http://github.com/hyunminkang/apigenome.

**Outline of the variant calling procedure**

The GotCloud vt pipeline detects variant sites and calls genotypes from a list of aligned sequence reads. Specifically, the pipeline consists of the following six key steps (see also the Figure). Most of these procedure will be integrated into GotCloud software package.

1. Variant detection: For each sequenced genome (in BAM/CRAMs), candidate variants are detected by vt discover2 software tools, separated by each chromosome. The candidate variants are normalized by vt normalize algorithm.
2. Variant consolidation : For each chromosome, the called variant sites are merged across the genomes, accounting for overlap of variants between genomes, using vt consolidate software tool.
3. Genotype and feature collection: For each 100kb chunk of genome, the genotyping module implemented in vt joint_genotype_sequential collects individual genotypes and variant features across the merged sites by iterating over sequenced genomes, focusing on the selected region.
4. Variant filtering: We use the inferred pedigree of related and duplicated samples to calculate Mendlian consistency statistics using vt milk-filter, and to train a variant quality classifier using a Support Vector Machine (SVM) implemented in the libsvm software package.

**Steps to prepare input files, install software and perform variant calling**

To produce variant calls using this pipeline, the following input files need to be prepared:

1. Aligned sequenced reads in BAM or CRAM format. Each BAM and CRAM file should contain one sample per subject. It also must be indexed using samtools index or equivalent software tools.
2. A sequence index file. Each line should contain [Sample ID] [Full Path to the BAM/CRAM file] [Contamination Estimates -- put zero if unknown]. See data/trio_data.index for example.
3. A pedigree file of nuclear families and duplicates in PED format. The pedigree file should contain only nuclear families. When a sample is duplicated, all Sample IDs representing the same individual (in the 2nd column) need to presented in a comma-separated way. In the 3rd and 4th column to represent their parents, only a representative sample ID is required. See data/trio_data.ped for example.

To clone and build the repository, follow these steps

$ git clone https://github.com/statgen/topmed_freeze4_calling.git

$ cd topmed_freeze4_calling

$ make # or make -j [numjobs] to expedite the process

$ wget ftp://anonymous@share.sph.umich.edu/gotcloud/ref/hs37d5-db142-v1.tgz # this will take a while

$ tar xzvf hs37d5-db142-v1.tgz

$ rm hs37d5-db142-v1.tgz

After these steps, modify scripts/gcconfig.pm to specify input data files or other parameters. Modifying the first section (index and ped file in particular) should be minimally required changes.

To perform variant discovery and consolidation, run the following step

$ perl scripts/step1-detect-and-merge-variants.pl [whitespace separated chromosome names to call]

After this step, follow the instruction to run make -f [Makefile] -j [numjobs] to complete the discovery tasks.

To genotype variants, run the following steps.

$ perl scripts/step2-joint-genotyping.pl [whitespace separated chromosome names to call]

After this step, following the instruction to run make -f [Makefile] -j [numjobs] to complete the genotyping tasks

To call X chromosome genotypes, the sex information is inferred by running the following steps

$ perl scripts/step2b-calc-sum-depth.pl

$ perl scripts/step2c-infer-sex.pl

After each step, following the instruction to run make -f [Makefile] -j [numjobs] to complete inferring sex.

To call chrX genotypes, run the following step.

$ perl scripts/step2x-joint-genotyping.pl X

To perform variant filtering using pedigree information, follow these steps.

$ perl scripts/step3a-compute-milk-score.pl [whitespace separated chromosome names to call] ## run makefile after this step

$ perl scripts/step3b-run-svm-milk-filter.pl [whitespace separated chromosome names to call]

$ perl scripts/step3c-run-milk-transfer.pl [whitespace separated chromosome names to call] ## this step is needed only when performing transfer learning from other chromosomes.

After all these steps, the called variant sites will be available at $(OUTPUT_DIR)/svm, and the genotypes will be available at $(OUTPUT_DIR)/paste.

For calling chrX, run the following step additionally

$ perl scripts/step3c-run-milk-transfer.pl X

**Variant Detection**

Variant detection from each sequenced (and aligned) genome is performed by vt discover2 software tool. The script step-1-detect-variants.pl provides a means to automate the variant detection across a large number of sequenced genomes.

The variant detection algorithm considers a variant as a potential candidate variant if there exists a mismatch between the aligned sequence reads and the reference genome. Because such a mismatch can easily occur by random errors, only potential candidate variants passing the following criteria are considered to be candidate variants in the next steps.

1. At least two identical evidences of variants must be observed from aligned sequence reads.
2. Each individual evidence will be normalized using the normalization algorithm implemented in vt normalize software tools.
3. Only evidence from the reads with mapping quality 20 or greater will be considered.
4. Duplicate reads, QC-failed reads, supplementary reads, secondary reads will be ignored.
5. Evidence of a variant within overlapping fragments of read pairs will not be double counted. Either end of the overlapping read pair will be soft-clipped using bam clipOverlap software tool.
6. Assuming per-sample heterozygosity of 0.1%, the posterior probability of having a variant at the position should be greater than 50%. This method is equivalent to how the glfSingle model is described.

The variant detection step is required only once per sequenced genome, when multiple freezes of variant calls are produced over the course of time.

**Variant Consolidation**

Variants detected from the discovery step are merged across all samples. This step is implemented in the step-2-detect-variants.pl scripts.

1. The non-reference alleles normalized by vt normalize algorithm are merged across the samples, and unique alleles are printed as biallelic candidate variants. The algorithm is published.
2. If there are alleles overlapping with other SNPs and Indels, overlap_snp and overlap_indel filters are added in the FILTER column of the corresponding variant.
3. If there are tandem repeats with 2 or more repeats with total repeat length of 6bp or longer, the variant is annotated as a potential VNTR (Variant Number Tandem Repeat), and overlap_vntr filters are added to the variant overlapping with the repeat track of the putative VNTR.

**Variant Genotyping and Feature Collection**

The genotyping step iterates all of the merged variant site over the sequenced samples. It iterates over BAM/CRAM files one at a time sequentially for each 1Mb chunk to perform contamination-adjusted genotyping and annotation of variant features for filtering. The following variant features are calculated during the genotyping procedure.

- AVGDP : Average read depth per sample
- AC : Non-reference allele count
- AN : Total number of alleles
- GC : Genotype count
- GN : Total genotype counts
- HWEAF : Allele frequency estimated from PL under HWE
- HWDAF : Genotype frequency estimated from PL under HWD
- IBC : [ Obs(Het) – Exp(Het) ] / Exp[Het]
- IS_IBC : [ Obs(Het) – Exp(Het) ] / Exp[Het] after correcting for population structure
- HWE_SLP : -log(HWE likelihood-ratio test p-value) ⨉ sign(IBC)
- IS_HWE_SLP : -log(HWE score test p-value after correcting for population structure) ⨉ sign(IS_IBC)
- ABE : Average fraction [#Ref Allele] across all heterozygotes
- ABZ : Z-score for tesing deviation of ABE from expected value (0.5)
- BQZ: Z-score testing association between allele and base qualities
- CYZ: Z-score testing association between allele and the sequencing cycle
- STZ : Z-score testing association between allele and strand
- NMZ : Z-score testing association between allele and per-read mismatches
- IOR : log [ Obs(non-ref, non-alt alleles) / Exp(non-ref, non-alt alleles) ]
- NM1 : Average per-read mismatches for non-reference alleles
- NM0 : Average per-read mismatches for reference alleles

The genotyping is done with adjustment for potential contamination. It uses adjusted genotype likelihood similar to the published method, but does not use estimated population allele frequency for the sake of computational efficiency. It conservatively assumes that the probability of observing a non-reference read given a homozygous reference genotype is equal to half of the estimated contamination level, (or 1%, whichever is greater). The probability of observing a reference read given a homozygous non-reference genotype is calculated in a similar way. This adjustment makes the heterozygous call more conservatively when the reference and non-reference allele reads are strongly imbalanced. For example, if 45 reference alleles and 5 non-reference alleles are observed at Q40, the new method calls it as homozygous reference genotype while the original method ignoring potential contamination calls it as heterozygous genotype. This adjustment improves the genotype quality of contaminated samples by reducing genotype errors by several fold.

**Variant Filtering**

The variant filtering in TOPMed Freeze 3 were performed by (1) first calculating Mendelian consistency scores using known familial relatedness and duplicates, and (2) training SVM classifier between the known variant sites (positive labels) and the Mendelian inconsistent variants (negative labels).

The negative labels are defined if the Bayes Factor for Mendelian consistency quantified as Pr(Reads | HWE, Pedigree) / Pr(Reads | HWD, no Pedigree) is less than 0.001. Also a variant is marked as negative labels if 3 or more samples show 20% of non-reference Mendelian discordance within families or genotype discordance between duplicated samples. The positive labels are the SNPs found polymorphic either in the 1000 Genomes Omni2.5 array or in HapMap 3.3, with additional evidence of being polymorphic from the sequenced samples. Variants eligible to be marked with both positive and negative labels are discarded from the labels. The SVM scores trained and predicted by the libSVM software tool are annotated in the VCF file.

Two additional hard filters were applied.

1. Excess heterozygosity filter (EXHET), if the Hardy-Weinberg disequilbrium p-value was less than 1e-6 in the direction of excess heterozygosity. An additional ~3,900 variants were filtered out by this filter.
2. Mendelian discordance filter (DISC), with 3 or more Mendelian inconsistencies or duplicate discordances observed from the samples. An additional ~370,000 variants were filtered out by this filter.

Functional annotation for each variant is provided in the INFO field using Pablo Cingolani’s snpEff 4.1 with a GRCh37.75 database. The current release includes only hard-call genotypes in the VCF files, without genotype likelihoods and with no missing genotypes. Genotype likelihoods may be included in future releases, at the cost of approximately 100x greater file size.

**Data Coordinating Center Methods**

Cathy Laurie, Bruce Weir and Ken Rice

Genetic Analysis Center, Department of Biostatistics, University of Washington

The following three approaches were used to identify and resolve sample identity issues.

**Concordance between annotated sex and biological sex inferred from the WGS data**

Biological sex was inferred from normalized X and Y chromosome depth for each sample (i.e. divided by autosomal depth) and from X chromosome heterozygosity. A small number of sex mismatches were detected as annotated females with low X and high Y chromosome depth or annotated males with high X and low Y chromosome depth. These samples were either excluded from the sample set to be released on dbGaP or their sample identities were resolved using information from array comparisons or pedigree checks. We also identified a small number of sex chromosome aneuploidies (XXY, XXX and mosaics such as XX/XO), which are annotated in file accompanying the genotypes, which has the suffix “sex_chromosome_karyotypes.txt”. This file provides the NWD ID, biological sex, and apparent sex chromosome karyotype of samples with inferred aneuploidies.

**Concordance between prior SNP array genotypes and WGS-derived genotypes**

Prior genome-wide SNP array data are available for 15 of the 19 accessions to be released in April 2017 (all except phs001024, phs001062, phs001032, and phs000997). The average percentage of individuals within the 15 accessions that have prior array data is 95%.

For six accessions, the prior array data analyzed for TOPMed were derived from ‘fingerprints’ compiled by dbGaP (Yumi Jin, see URL below). The fingerprints consist of genotypes from a set of 10,000 bi-allelic autosomal SNP markers chosen to occur on multiple commercial arrays and to have a minor allele frequency (MAF) > 5%. For the remaining nine accessions, all autosomal SNPs with MAF > 5% on a genome-wide array were used. In both cases (fingerprints and full array), percent concordance was determined by matching on heterozygous versus homozygous status (rather than specific alleles) to avoid strand issues. Concordance percentages for array-WGS matches were generally in the high 90s, while those considered to be mismatches were in the 50-60% range (empirically determined to be the expected matching level for random pairs of samples). We found that 99.8% of the 14,412 WGS samples tested were concordant with prior array data. Discordant samples were either excluded from the April 2017 release or resolved as sample switches using pedigree and/or sex-mismatch results.

SNP fingerprints: http://www.ashg.org/2014meeting/abstracts/fulltext/f140122979.htm

**Comparisons of observed and expected relatedness from pedigrees**

Kinship coefficients (KCs) were estimated for all pairs of individuals using ~250k single nucleotide variants that are autosomal, MAF >5%, and pruned to have low linkage disequilibrium (r2<0.1) with one another. The estimation procedure used ‘PC-Relate’ (Conomos et al. 2016, DOI: 10.1016/j.ajhg.2015.11.022), which is robust to population structure, admixture and departures from Hardy-Weinberg Disequilibrium. The KC estimates were compared to those expected from pedigrees for the accessions with annotated family structure (phs000956, phs000974, phs000988, phs000964, phs000954 , and phs001143). Discrepancies between observed and expected KCs were investigated and, in many cases, resolved either by correcting sample-subject mapping for sample switches or by making a change in the pedigree structure. Pedigree changes were warranted when one alteration resolved multiple KC discrepancies or when supported by additional information from the studies.
