# Supplementary material for: Common α-globin variants modify hematologic and other clinical phenotypes in sickle cell trait and disease
Source: PLoS Genet. 2018 Mar 28;14(3):e1007293. doi: 10.1371/journal.pgen.1007293 (PMC5891078; doi:10.1371/journal.pgen.1007293)
Supplement: S2 File — Published with permission of the TOPMed Publications Committee. (DOCX) [file pgen.1007293.s011.docx]

**S2 File. The Trans-Omics in Precision Medicine Program (TOPMed).**

Published with permission of the TOPMed Publications Committee.

| **Name** | **Institution(s)** |
| --- | --- |
| Abe, Namiko | New York Genome Center |
| Abecasis, Goncalo | University of Michigan |
| Allred, Nicholette (Nichole) Palmer | Wake Forest Baptist Health |
| Almasy, Laura | Children's Hospital of Philadelphia, University of Pennsylvania |
| Ament, Seth | University of Maryland |
| Anderson, Peter | University of Washington |
| Anugu, Pramod | University of Mississippi |
| Applebaum-Bowden, Deborah | National Institutes of Health |
| Arnett, Donna K | University of Kentucky |
| Ashley-Koch, Allison | Duke University |
| Aslibekyan, Stella | University of Alabama |
| Assimes, Tim | Stanford University |
| Auer, Paul | University of Wisconsin Milwaukee |
| Avramopoulos, Dimitrios | Johns Hopkins University |
| Barnes, Kathleen | University of Colorado at Denver |
| Barr, R. Graham | Columbia University |
| Barron-Casella, Emily | Johns Hopkins University |
| Beaty, Terri | Johns Hopkins University |
| Becker, Diane | Johns Hopkins University |
| Becker, Lewis | Johns Hopkins University |
| Beer, Rebecca | NIH National Heart, Lung, and Blood Institute |
| Begum, Ferdouse | Johns Hopkins University |
| Beitelshees, Amber | University of Maryland |
| Bezerra, Marcos | Fundação de Hematologia e Hemoterapia de Pernambuco - Hemope |
| Bielak, Larry | University of Michigan |
| Blackwell, Thomas | University of Michigan |
| Blangero, John | University of Texas Rio Grande Valley School of Medicine |
| Boerwinkle, Eric | University of Texas Health |
| Borecki, Ingrid | University of Washington |
| Bowler, Russell | National Jewish Health |
| Broeckel, Ulrich | Medical College of Wisconsin |
| Bunting, Karen | New York Genome Center |
| Burchard, Esteban | University of California, San Francisco |
| Cardwell, Jonathan | University of Colorado at Denver |
| Carlson, Sara | University of Washington |
| Carty, Cara | Women's Health Initiative |
| Casaburi, Richard | University of California, Los Angeles |
| Casella, James | Johns Hopkins University |
| Chang, Christy | University of Maryland |
| Chen, Bo-Juen | New York Genome Center |
| Chen, Wei-Min | University of Virginia |
| Chen, Yii-Der Ida | Los Angeles Biomedical Research Institute |
| Cho, Michael | Brigham & Women's Hospital |
| Chuang, Lee-Ming | National Taiwan University |
| Cornell, Elaine | University of Vermont |
| Correa, Adolfo | University of Mississippi |
| Crandall, Carolyn | University of California, Los Angeles |
| Crapo, James | National Jewish Health |
| Cupples, L Adrienne | Boston University |
| Curran, Joanne | University of Texas Rio Grande Valley School of Medicine |
| Curtis, Jeffrey | University of Michigan |
| Custer, Brian | Blood Systems Research Institute UCSF |
| Damcott, Coleen | University of Maryland |
| David, Sean | Stanford University |
| Davis, Colleen | University of Washington |
| de Andrade, Mariza | Mayo Clinic |
| DeBaun, Michael | Vanderbilt University |
| Deka, Ranjan | University of Cincinnati |
| DeMeo, Dawn | Brigham & Women's Hospital |
| Devine, Scott | University of Maryland |
| Do, Ron | Icahn School of Medicine at Mount Sinai |
| Duan, Qing | University of North Carolina |
| Duggirala, Ravi | University of Texas Rio Grande Valley School of Medicine |
| Durda, Peter | University of Vermont |
| Dutcher, Susan | Washington University in St Louis |
| Eaton, Charles | Brown University |
| Ekunwe, Lynette | University of Mississippi |
| Ellinor, Patrick | Massachusetts General Hospital |
| Farber, Charles | University of Virginia |
| Farnam, Leanna | Brigham & Women's Hospital |
| Fingerlin, Tasha | National Jewish Health |
| Fornage, Myriam | University of Texas Health |
| Franceschini, Nora | University of North Carolina |
| Fu, Mao | University of Maryland |
| Gabriel, Stacey | The Broad Institute |
| Gan, Weiniu | National Institutes of Health |
| Gao, Yan | University of Mississippi |
| Gass, Margery | Fred Hutchinson Cancer Research Center |
| Germer, Soren | New York Genome Center |
| Gladwin, Mark | University of Pittsburgh |
| Glahn, David | Yale University |
| Gong, Da-Wei | University of Maryland |
| Goring, Harald | University of Texas Rio Grande Valley School of Medicine |
| Gu, C. Charles | Washington University in St Louis |
| Guan, Yue | University of Maryland |
| Guo, Xiuqing | Los Angeles Biomedical Research Institute |
| Haessler, Jeff | Fred Hutchinson Cancer Research Center, Women's Health Initiative |
| Hall, Michael | University of Mississippi |
| Harris, Daniel | University of Maryland |
| Hawley, Nicola | Yale University |
| He, Jiang | Tulane University |
| Heckbert, Susan | University of Washington |
| Hernandez, Ryan | University of California, San Francisco |
| Herrington, David | Wake Forest Baptist Health |
| Hersh, Craig | Brigham & Women's Hospital |
| Hidalgo, Bertha | University of Alabama |
| Hixson, James | University of Texas Health |
| Hokanson, John | University of Colorado at Denver |
| Hong, Elliott | University of Maryland |
| Hoth, Karin | University of Iowa |
| Hsiung, Chao (Agnes) | National Health Research Institute Taiwan |
| Huston, Haley | Blood Works Northwest |
| Hwu, Chii Min | Taichung Veterans General Hospital Taiwan |
| Irvin, Marguerite Ryan | University of Alabama |
| Jackson, Rebecca | Ohio State University Wexner Medical Center |
| Jaquish, Cashell | National Institutes of Health |
| Jhun, Min A | University of Michigan |
| Johnsen, Jill | Blood Works Northwest, University of Washington |
| Johnson, Andrew | NIH National Heart, Lung, and Blood Institute |
| Johnson, Craig | University of Washington |
| Jones, Kimberly | Johns Hopkins University |
| Kaplan, Robert | Albert Einstein College of Medicine |
| Kardia, Sharon | University of Michigan |
| Kathiresan, Sekar | The Broad Institute |
| Kaufman, Laura | Brigham & Women's Hospital |
| Kelly, Shannon | Blood Systems Research Institute UCSF |
| Kenny, Eimear | Icahn School of Medicine at Mount Sinai |
| Kessler, Michael | University of Maryland |
| Kinney, Greg | University of Colorado at Denver |
| Konkle, Barbara | Blood Works Northwest |
| Kooperberg, Charles | Fred Hutchinson Cancer Research Center |
| Kramer, Holly | Loyola University |
| Krauter, Stephanie | University of Washington |
| Lange, Christoph | Harvard School of Public Health |
| Lange, Ethan | University of North Carolina |
| Lange, Leslie | University of Colorado at Denver |
| Laurie, Cathy | University of Washington |
| LeBoff, Meryl | Brigham & Women's Hospital |
| Lee, Wen-Jane | Taichung Veterans General Hospital Taiwan |
| Levy, Dan | NIH National Heart, Lung, and Blood Institute, National Institutes of Health |
| Lewis, Joshua | University of Maryland |
| Li, Yun | University of North Carolina |
| Liu, Simin | Brown University, Women's Health Initiative |
| Liu, Yongmei | Wake Forest Baptist Health |
| Locke, Adam | McDonnell Genome Institute |
| Loos, Ruth | Icahn School of Medicine at Mount Sinai |
| Luo, James | NIH National Heart, Lung, and Blood Institute, National Institutes of Health |
| Mahaney, Michael | University of Texas Rio Grande Valley School of Medicine |
| Make, Barry | Johns Hopkins University |
| Manichaikul, Ani | University of Virginia |
| Manson, JoAnn | Brigham & Women's Hospital |
| Martin, Lisa | George Washington University |
| Mathai, Susan | University of Colorado at Denver |
| Mathias, Rasika | Johns Hopkins University |
| McArdle, Patrick | University of Maryland |
| McDonald, Merry-Lynn | University of Alabama |
| McFarland, Sean | Harvard University |
| McGarvey, Stephen | Brown University |
| Mei, Hao | University of Mississippi |
| Meyers, Deborah A | University of Arizona |
| Mikulla, Julie | National Institutes of Health |
| Min, Nancy | University of Mississippi |
| Minear, Mollie | National Institutes of Health |
| Minster, Ryan L | University of Pittsburgh |
| Mitchell, Braxton | University of Maryland |
| Montasser, May E. | University of Maryland |
| Musani, Solomon | University of Mississippi |
| Mwasongwe, Stanford | University of Mississippi |
| Mychaleckyj, Josyf C | University of Virginia |
| Nadkarni, Girish | Icahn School of Medicine at Mount Sinai |
| Naik, Rakhi | Johns Hopkins University |
| Natarajan, Pradeep | The Broad Institute, Harvard University, Massachusetts General Hospital |
| Nekhai, Sergei | Howard University |
| Nickerson, Deborah | University of Washington |
| North, Kari | University of North Carolina |
| O'Connell, Jeff | University of Maryland |
| O'Connor, Tim | University of Maryland |
| Ochs-Balcom, Heather | University at Buffalo |
| Pankow, James | University of Minnesota |
| Papanicolaou, George | National Institutes of Health |
| Parker, Margaret | Brigham & Women's Hospital |
| Parsa, Afshin | University of Maryland |
| Pattison, Jessica Tangarone | University of Michigan |
| Penchev, Sara | National Jewish Health |
| Perez, Marco | Stanford University |
| Perry, James | University of Maryland |
| Peters, Ulrike | Fred Hutchinson Cancer Research Center, University of Washington |
| Peyser, Patricia | University of Michigan |
| Phillips, Larry | Emory University |
| Phillips, Sam | University of Washington |
| Pollin, Toni | University of Maryland |
| Post, Wendy | Johns Hopkins University |
| Powers Becker, Julia | University of Colorado at Denver |
| Preuss, Michael | Icahn School of Medicine at Mount Sinai |
| Prokopenko, Dmitry | Harvard University |
| Psaty, Bruce | University of Washington |
| Qasba, Pankaj | National Institutes of Health |
| Qiao, Dandi | Brigham & Women's Hospital |
| Raffield, Laura edit | University of North Carolina |
| Ramachandran, Vasan | Boston University |
| Rao, D.C. | Washington University in St Louis |
| Rasmussen-Torvik, Laura | Northwestern University |
| Ratan, Aakrosh | University of Virginia |
| Redline, Susan | Brigham & Women's Hospital |
| Reed, Robert | University of Maryland |
| Regan, Elizabeth | National Jewish Health |
| Reiner, Alex | University of Washington |
| Rice, Ken | University of Washington |
| Rich, Stephen | University of Virginia |
| Rotter, Jerome | Los Angeles Biomedical Research Institute |
| Russell, Pamela | University of Colorado at Denver |
| Ruuska, Sarah | Blood Works Northwest |
| Ryan, Kathy | University of Maryland |
| Sakornsakolpat, Phuwanat | Brigham & Women's Hospital |
| Salimi, Shabnam | University of Maryland |
| Sandow, Kevin | Los Angeles Biomedical Research Institute |
| Sankaran, Vijay | Harvard University |
| Schwander, Karen | Washington University in St Louis |
| Schwartz, David | University of Colorado at Denver |
| Sciurba, Frank | University of Pittsburgh |
| Sheehan, Vivien | Baylor College of Medicine |
| Shetty, Amol | University of Maryland |
| Sheu, Wayne Hui-Heng | Taichung Veterans General Hospital Taiwan |
| Silver, Brian | University of Massachusetts Memorial Health Center |
| Silverman, Edwin | Brigham & Women's Hospital |
| Smith, Jennifer | University of Michigan |
| Smith, Josh | University of Washington |
| Smith, Tanja | New York Genome Center |
| Smoller, Sylvia | Albert Einstein College of Medicine |
| Snively, Beverly | Wake Forest Baptist Health |
| Sotoodehnia, Nona | University of Washington |
| Streeten, Elizabeth | University of Maryland |
| Sung, Yun Ju | Washington University in St Louis |
| Sylvia, Jody | Brigham & Women's Hospital |
| Sztalryd, Carole | University of Maryland |
| Tang, Hua | Stanford University |
| Taylor, Kent | Los Angeles Biomedical Research Institute |
| Taylor, Simeon | University of Maryland |
| Telen, Marilyn | Duke University |
| Tinker, Lesley | Women's Health Initiative |
| Tirschwell, David | University of Washington |
| Tiwari, Hemant | University of Alabama |
| Tracy, Russell | University of Vermont |
| Tsai, Michael | University of Minnesota |
| Vaidya, Dhananjay | Johns Hopkins University |
| Walker, Tarik | University of Colorado at Denver |
| Wallace, Robert | University of Iowa |
| Walts, Avram | University of Colorado at Denver |
| Wan, Emily | Brigham & Women's Hospital |
| Watson, Karol | University of California, Los Angeles |
| Weeks, Daniel E. | University of Pittsburgh |
| Weir, Bruce | University of Washington |
| Weiss, Scott | Brigham & Women's Hospital |
| Williams, Kayleen | University of Washington |
| Williams, L. Keoki | Henry Ford Health System |
| Wilson, Carla | Brigham & Women's Hospital |
| Wilson, James | University of Mississippi |
| Xu, Huichun | University of Maryland |
| Yanek, Lisa | Johns Hopkins University |
| Yang, Ivana | University of Colorado at Denver |
| Yang, Rongze | University of Maryland |
| Zaghloul, Norann | University of Maryland |
| Zhang, Yingze | University of Pittsburgh |
| Zhao, Snow Xueyan | National Jewish Health |
| Zhao, Wei | University of Michigan |
| Zhi, Degui | University of Texas Health |
| Zody, Michael | New York Genome Center |

**Hematology & Hemostasis TOPMed Working Group**

Laura Almasy, Allison Ashley-Koch, Paul Auer, Emily Barron-Casella, Lewis Becker, John Blangero, Michael Bowers. Jennifer Brody, Pamela Burton, James Casella, Ming-Huei Chen, Michael Cho, Adolfo Correa, Paul de Vries, Qing Duan, John Eicher, Ugur Erickson, Nauder Faraday, James Floyd, Santhi Ganesh, Manjit Hanspal, Craig Hersh, Jennifer Huffman, Deepti Jain, Nancy Jenny, Jill Johnsen, Andrew Johnson, Shannon Kelly, Malgorzata Klauzinska, Barbara Konkle, Charles Kooperberg, Jacquelyn Lane, Leslie Lange, Ethan Lange, Cathy Laurie, Cecelia Laurie, Grace Lee, Guillaume Lettre, David Levine, Joshua Lewis, Yun Li, Bingshan Li, Rasika Mathias, Braxton Mitchell, Elizabeth Mollica, Alanna C Morrison, Rakhi Naik, Deborah Nickerson, Jeff O'Connell, Christopher O'Donnell, Nathan Pankratz, Linda Polfus, Bruce Psaty, Laura Raffield, Alex Reiner, Erin Rice, Stephen Rich, Shabnam Salimi, Jenny Schoenberg, Nicholas Smith, Ci Song, Adrienne Stilp, Hua Tang, Margaret Taub, Marilyn Telen, Timothy A. Thornton, Russell Tracy, Kate Wehr, Ellen Werner, Marsha Wheeler, Ann Whitney, James Wilson, Lisa Yanek, Yu-Chung Yang, Xiuwen Zheng

**Structural Variation TOPMed Working Group**

Michael Bowers, Ulrich Broeckel, Lavanya Challagundla, Kei Hang Katie Chan, Bradley Coe, Scott Devine, Evan Eichler, Leslie Emery, Alex Garcia Lugo, Stephanie Gogarten, C. Charles Gu, Yuelong (John) Guo, Ira Hall, Bob Handsaker, James Hixson, Deepti Jain, Jill Johnsen, Priyanka Joshi, Goo Jun, Hyun Min Kang, Charles Kooperberg, Cathy Laurie, Seung-been Lee, David Levine, Honghuang Lin, Simin Liu, Rasika Mathias, Steve McCarroll, Giuseppe Narzisi, Deborah Nickerson, Jeff O'Connell, Nathan Pankratz, Emmanuel Peprah, Aaron Quinlan, Aakrosh Ratan, Alex Reiner, Erin Rice, Ingo Ruczinski, Aniko Sabo, William Salerno, Jenny Schoenberg, Vinodh Srinivasasainagendra, Margaret Taub, Tychele Turner, Kate Wehr, Sandy Zellner, Degui Zhi, Sebastian Zoellner

**Diabetes TOPMed Working Group**

Goncalo Abecasis, Nicholette (Nichole) Palmer Allred, Marcio Almeida, Donna K Arnett, Danika Baez, Rebecca Beer, Larry Bielak, John Blangero, Mike Boehnke, Eric Boerwinkle, Michael Bowers, Camille Breaux, Jennifer Brody, Brian Cade, Jin Choul Chai, Kei Hang Katie Chan, Yii-Der Ida Chen, Yen-Feng Chiu, Ren-Hua Chung, Mete Civelek, Adolfo Correa, Coleen Damcott, Paul de Vries, Josee Dupuis, Ugur Erickson, Irene Felicetti, Jason Flannick, Jose Florez, James Floyd, Amanda Fretts, Mao Fu, Anna Furniss, Alex Garcia Lugo, Kyle Gaulton, Da-Wei Gong, Mark Goodarzi, Xiuqing Guo, Jiang He, Bertha Hidalgo, Heather Highland, Marie-France Hivert, Deepti Jain, Michelle Jones, Kizer Jorge, Priyanka Joshi, Rita Kalyani, Robert Kaplan, Dawn Keene, Tanika Kelly, Greg Kinney, Erica Kleinbrink, Charles Kooperberg, Jacquelyn Lane, Leslie Lange, Wen-Jane Lee, I-Te Lee, Rozenn Lemaitre, Samantha Lent, Aaron Leong, Xiaochen Lin, Simin Liu, Ching-Ti Liu, Qing Liu, Ruth Loos, Anubha Mahajan, Alisa Manning, JoAnn Manson, Rasika Mathias, Mark McCarthy, Stephen McGarvey, John McLenithan, James Meigs, Braxton Mitchell, Karen Mohlke, Elizabeth Mollica, May E. Montasser, Jee-Young Moon, Andrew Morris, Alanna C Morrison, Rakhi Naik, Rami Nassir, Maggie Ng, Jeff O'Connell, James Pankow, James Perry, Patricia Peyser, Larry Phillips, Toni Pollin, Bianca Porneala, Qibin Qi, Laura Raffield [edit], Vasan Ramachandran, Laura Rasmussen-Torvik, Susan Redline, Michelle Reid, Erin Rice, Stephen Rich, Jonathan Roskos, Jerome Rotter, Emily Russell, Michele Sale, Shabnam Salimi, Chloe Sarnowski, Richa Saxena, Bonnie L Schoenbein, Jenny Schoenberg, Mark Seielstad, Elizabeth Selvin, Aladdin Shadyab, Wayne Hui-Heng Sheu, Edwin Silverman, Robert Sladek, Soren Snitker, Tamar Sofer, Marla Spires, Carole Sztalryd, Simeon Taylor, Kent Taylor, Daune Thorington, Heming Wang, Tao Wang, Kate Wehr, Jennifer Wessel, Peggy White, James Wilson, Quenna Wong, Peitao Wu, Huichun Xu, Lisa Yanek, Rongze Yang, Norann Zaghloul, Isabel Zhang
